# Supplementary material for: Overhauser enhanced liquid state nuclear magnetic resonance spectroscopy in one and two dimensions
Source: Nat Commun. 2024 Jul 13;15:5904. doi: 10.1038/s41467-024-50265-5 (PMC11246421; doi:10.1038/s41467-024-50265-5)
Supplement: Supplementary file 1 — Supplementary Information [file 41467_2024_50265_MOESM1_ESM.pdf]

Supplementary Information

Overhauser enhanced liquid state nuclear magnetic resonance spectroscopy in one and two dimensions

Marcel Levien[1,2,§], Luming Yang[1], Alex van der Ham[1], Maik Reinhard[1,2], Michael John[3], Armin Purea[4], Jürgen Ganz[4], Thorsten Marquardsen[4,‡], Igor Tkach[1], Tomas Orlando[1†], Marina Bennati[1,2*]

[1]Electron-Spin Resonance Spectroscopy, Max Planck Institute for Multidisciplinary Sciences, Am Fassberg 11, 37077 Göttingen, Germany.

[2]Institute of Physical Chemistry, Department of Chemistry, Georg-August-University, Tammannstr. 6, 37077 Göttingen, Germany.

[3]Institute of Organic and Biomolecular Chemistry, Department of Chemistry, Georg-August-University, Tammannstr. 2, 37077 Göttingen, Germany.

[4]Bruker Biospin GmbH, Rudolf-Plank-Str. 23, 76275 Ettlingen, Germany.

[§]Present Address: Institut des Sciences et Ingénierie Chimiques, École Polytechnique Fédérale de Lausanne (EPFL), CH-1015 Lausanne, Switzerland.

[†]Present Address: National High Magnetic Field Laboratory, 1800 E. Paul Dirac Dr., 32310 Tallahassee, Florida, USA.

[‡]Deceased

*Correspondence to: marina.bennati@mpinat.mpg.de

Contents

Supplementary equations 1: Overhauser Theory 2
Supplementary equations 2: Saturation factor 5
Supplementary Note: 1: Characterization of the liquid state DNP probe 7
Supplementary Note: 2: Characterization of the frequency agile gyrotron 12
Supplementary Note 3: 1D DNP NMR spectra in non-polar and polar solvents 14
Supplementary Note 4: DNP enhanced 2D ^{13}C-^{13}C correlation NMR experiments 18
Supplementary Discussion 1: Electron spin relaxation times at 263 GHz, room temperature, and experimental saturation factor 24
Supplementary Discussion 2: Mechanistic interpretation of the aromatic ^{13}C enhancements at 9.4 Tesla 30
Supplementary Discussion 3: Sensitivity comparison of 1D and 2D DNP with commercial probes 36
Supplementary References 43

Supplementary equations 1: Overhauser Theory

In liquids, spin polarization between electron and nuclear spins can be transferred via the Overhauser effect, which is based on cross-relaxation and driven by a modulation of the hyperfine (hf) interaction between the coupled spins.[1-3]

At thermal equilibrium, the polarization of a nuclear spin $I = ½$ is given by:[4]

$$P = \tanh\left(\frac{g_n \mu_N B_0}{2k_B T}\right) \tag{1}$$

where P is the polarization, g_n the g factor of the nucleus, μ_N the nuclear magneton, B_0 the external magnetic field, k_B the Boltzmann factor, and T the absolute temperature. The Overhauser effect is described by considering the four energy levels of an electron spin $S = ½$ coupled to a nuclear spin $I = ½$ (e.g. ^1H or ^{13}C) and the transition probabilities $w_{0,1,2}$ between pairs of states labelled according to Supplementary Figure 1a. For constant microwave pumping on resonance with one electron spin transition, the Overhauser equation for the steady-state in liquids is expressed as:[2]

$$\varepsilon = {\langle I_z \rangle}/{I_0} = 1 - \frac{w_2 - w_0}{w_0 + 2w_1 + w_2} \cdot \frac{w_0 + 2w_1 + w_2}{w_0 + 2w_1 + w_2 + R_{1\mathrm{dia}}} \cdot s \cdot \frac{|\gamma_e|}{\gamma_n} \tag{2}$$

$$= 1 - \xi \cdot f \cdot s \cdot \frac{|\gamma_e|}{\gamma_n}$$

Here, ε is the NMR signal enhancement that is proportional to the nuclear spin expectation value $\langle I_z \rangle$, I_0 is the nuclear Boltzmann polarization, γ_e and γ_n are the gyromagnetic ratios of electron and nuclear spins, respectively. $R_{1\mathrm{dia}}$ is the diamagnetic contribution to the total nuclear-spin relaxation rate.

The terms ξ and f are called coupling and leakage factors, respectively; s is the effective saturation factor of the radical, which takes into account the saturation of the pumped EPR transition plus the cross saturation between EPR hf lines, called ELDOR effect.[5,6] A brief derivation of s is given in Supplementary equations 2. The NMR signal enhancement is proportional to the enhanced polarization of the nucleus and is experimentally accessed by the measurement of the NMR signal with and without microwave irradiation (see Methods).

The leakage factor considers the nuclear relaxation that does not occur through interaction with the electron spin. It is experimentally accessed by comparison of the nuclear longitudinal relaxation time in the presence (T_{1n}) and absence (T_{1n}^0) of polarizing agent:

$$f = 1 - \frac{T_{1n}}{T_{1n}^0} \tag{3}$$

Finally, the coupling factor contains information on the cross-relaxation between electron and nuclear spins. The transition probabilities w_0 and w_2 depend on the stochastic modulation of the hf coupling through molecular motions. Translational and rotational diffusion usually modulate the dipolar (anisotropic) part of the hf coupling, while molecular collisions modulate the scalar (isotropic) component. In the liquid state, the contribution of dipolar and scalar hf relaxation can be expressed through additive terms in the coupling factor:[2]

$$\xi = \frac{w_2^{\mathrm{dip}} - w_0^{\mathrm{dip}} - w_0^{\mathrm{sc}}}{w_0^{\mathrm{dip}} + 2w_1^{\mathrm{dip}} + w_2^{\mathrm{dip}} + w_0^{\mathrm{sc}}} \tag{4}$$

where the superscripts refer to the different scalar and dipolar mechanisms, respectively. The relations between probabilities $w_{0,1,2}$ and spectral densities for a fast, randomly fluctuating hf Hamiltonian were derived by Solomon and can be expressed as:[3]

$$w_0 = w_0^{\mathrm{dip}} + w_0^{\mathrm{sc}} = k_{\mathrm{dip}} \cdot J(\omega_\mathrm{n} - \omega_\mathrm{e}, \tau_{\mathrm{dip}}) + k_{\mathrm{sc}} \cdot J(\omega_\mathrm{n} - \omega_\mathrm{e}, \tau_{\mathrm{sc}})$$

$$w_{1,\mathrm{n}} = {}^3/_2\, k_{\mathrm{dip}}\, J(\omega_\mathrm{n}, \tau_{\mathrm{dip}})$$

$$w_2 = 6k_{\mathrm{dip}}\, J(\omega_\mathrm{n} + \omega_\mathrm{e}, \tau_{\mathrm{dip}}) \tag{5}$$

where $J(\omega, \tau)$ is the spectral density function, τ_{dip} and τ_{sc} are the correlation times for dipolar and scalar relaxation, ω_e and ω_n the electron and nuclear Larmor frequencies, k_{dip} and k_{sc} are pre-factors that contain the mean squared fluctuation of dipolar and scalar interactions, respectively. With the approximation $(\omega_\mathrm{e} \pm \omega_\mathrm{n}) \approx \omega_\mathrm{e}$, the coupling factor can be reformulated as a function of spectral densities:

$$\xi = \frac{5k_{\mathrm{dip}} \cdot J_{\mathrm{dip}}(\omega_\mathrm{e}, \tau_{\mathrm{dip}}) - k_{\mathrm{sc}} \cdot J_{\mathrm{sc}}(\omega_\mathrm{e}, \tau_{\mathrm{sc}})}{7k_{\mathrm{dip}} \cdot J_{\mathrm{dip}}(\omega_\mathrm{e}, \tau_{\mathrm{dip}}) + 3k_{\mathrm{dip}} \cdot J_{\mathrm{dip}}(\omega_\mathrm{n}, \tau_{\mathrm{dip}}) + k_{\mathrm{sc}} \cdot J_{\mathrm{sc}}(\omega_\mathrm{e}, \tau_{\mathrm{sc}})} \tag{6}$$

For translational diffusion, we employ the spectral density of the force free hard spheres model introduced by Freed and co-workers,[7,8] which assumes that the paramagnetic species are in the center of hard spherical molecules:

$$J_{\mathrm{dip}}(\omega, \tau_{\mathrm{dip}}) = J_D(\omega, \tau_\mathrm{D}) = \frac{1 + \frac{5z}{8} + \frac{z^2}{8}}{1 + z + \frac{z^2}{2} + \frac{z^3}{6} + \frac{4z^4}{81} + \frac{z^5}{81} + \frac{z^6}{648}} \tag{7}$$

with $z = \sqrt{2\omega\tau_\mathrm{D}}$ and $\tau_{\mathrm{dip}} = \tau_\mathrm{D} = \frac{r_\mathrm{D}^2}{D_\mathrm{s} + D_{\mathrm{r},}}$, where r_D is the point-dipole distance of closest approach between electron and nuclear spins, D_s and D_r are the local diffusion coefficient of the target and the PA in the given solvent, respectively. The pre-factors k_{dip} and k_{sc} were given in ref.[9].

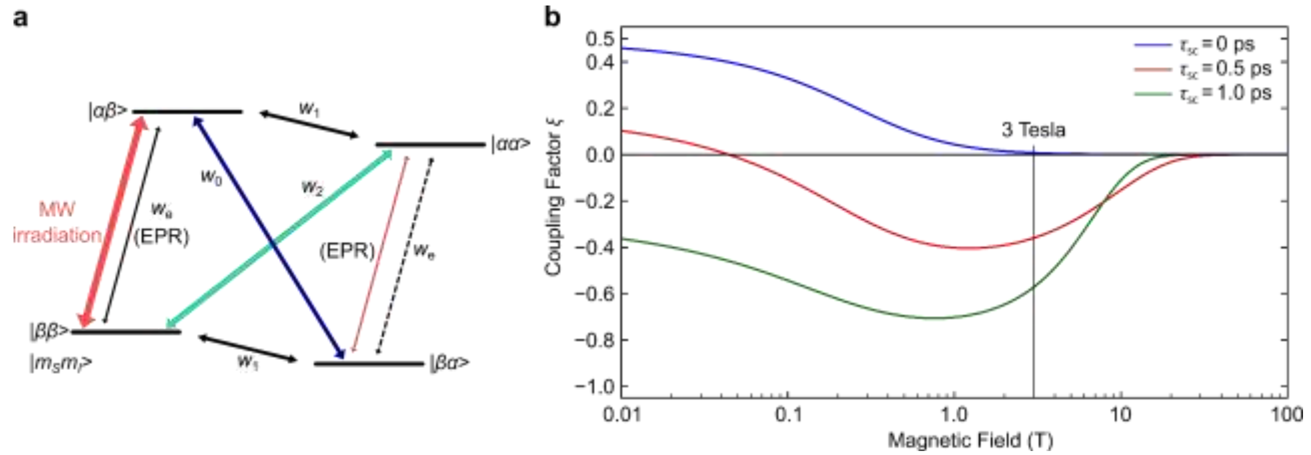

Supplementary Figure 1: (a) Energy level diagram of an electron spin coupled to a nuclear spin-½ (with $|m_S,m_I>$). Here, w_2 (green arrow), w_0 (blue arrow), w_e (black, thin solid and dashed arrows) and w_1 (black, think solid arrows) are the double-quantum, zero-quantum, electron, and nuclear transition probabilities per unit time, respectively. MW irradiation indicated with red arrows. (b) Calculated coupling factor using eq. 4-9 as a function of the external magnetic field for two individual type of collisions (red and green with $\tau_{sc} = 0.5$ ps and $\tau_{sc} = 1.0$ ps, respectively, $F = \frac{\langle A_i \rangle^2}{\hbar^2 \tau_p} = 1.69 \times 10^{24}$ rad^2/s^2) and without scalar contribution (blue, purely dipolar). dipolar contribution was assumed to be caused by translational diffusion with $\tau_D = 114$ ps ($D = 1.4 \times 10^{-9}$ m^2/s, $r_D = 4.0$ Å), $k_D = 0.0788$, and $c = 25$ mM. The simulation parameters were adopted from ref.[9], where they were used to fit the ^{13}C magnetic field dependence of CCl_4 using TEMPONE-^{15}N-d_{16} as a PA.

It is important to realize that $w_{0,2}^{\mathrm{dip}}$ have mostly decayed at an external magnetic field $\gtrsim 3$ Tesla (Supplementary Figure 1b), because the correlation time of translational diffusion τ_D is on the order of 20 – 200 ps[9] and thus dipolar relaxation contributes only with w_1^{dip} to the denominator of the coupling factor as:

$$\xi_{\text{high field}} \approx -\, w_0^{\mathrm{sc}} / \left(2 w_1^{\mathrm{dip}} + w_0^{\mathrm{sc}}\right) \quad (8)$$

Therefore, the coupling factors observed at high fields are determined by intermolecular scalar cross-relaxation.[1] To describe the modulation of the isotropic hf coupling through molecular collisions, we have adopted the so-called pulse model introduced by Müller-Warmuth and co-workers.[10,11] Assuming that the time evolution of the hf coupling during a molecular collision (called pulse) can be approximated by Lorentzian line shapes of width (duration) $2\tau_{sc}$, the spectral density can be given analytically as:

$$J_{sc}(\omega_e, \tau_{sc}) = \sum_i \frac{4\pi^2 \langle A_{\mathrm{iso},i} \rangle^2}{\tau_{\mathrm{p},i}} \left[\tau_{\mathrm{sc},i} \exp\left(-\omega_e \tau_{\mathrm{sc},i}\right)\right]^2 \quad (9)$$

with $\langle A_{\mathrm{iso},i} \rangle$ the average amplitude isotropic hf coupling, $\frac{1}{\tau_{\mathrm{p},i}}$ the frequency, and $2\tau_{\mathrm{sc},i}$ the duration of the collision *i*. As an example, neglecting rotational diffusion,[12,13] we have simulated the coupling factor in

Supplementary Figure 1b as a function of the magnetic field by employing spectral densities for translational diffusion and molecular collision (eq. 6, 7, 9).

Supplementary equations 2: Saturation factor

The saturation factor in the Overhauser equation (eq. 2) quantifies the degree to which the EPR resonance of the polarizing agent (PA) is saturated by microwave irradiation, and is generally formulated as:

$$s = \frac{S_0 - \langle S_z \rangle}{S_0} \tag{10}$$

where $\langle S_z \rangle$ is the expectation value of the electron spin under MW irradiation and S_0 is the value at thermal equilibrium, respectively.

In case of a single EPR resonance line of a spin $S = {}^1\!/_2$, the saturation factor is derived from the Bloch equations:[14]

$$s = 1 - \frac{1}{1 + \gamma_e^2 T_{1e} T_{2e} B_{1e}^2} \tag{11}$$

Here, B_{1e} is the microwave field amplitude, T_{1e} and T_{2e} the electron spin lattice and spin-spin relaxation times, respectively. If the EPR resonance is split by hf coupling to a nearby nuclear spin, as in the case of a nitroxide radical, the so-called ELDOR effect transfers saturation among hf EPR lines and the saturation factor becomes the mean value of the saturation factor of each hf transition s_i:

$$s_{\mathrm{eff}} = \frac{1}{n} \sum_{i=1}^{n} s_i \tag{12}$$

Here, n is the number of EPR hf lines and s_i the saturation factor of each transition. For the case of one electron spin $S = {}^1\!/_2$ coupled to one nucleus $I = {}^1\!/_2$, e.g. ^{15}N labelled nitroxide, we previously derived analytical solutions for the saturation factor of both hf lines when pumping one line (labeled line 1) as:[5]

$$s_{1,\mathrm{pump}}(B_{1e}) = 1 - \frac{w_e[2(w_e + w_n) + cK_X]}{\frac{1}{4}\gamma_e^2 B_{1e}^2 T_{2e}(4w_e + 2w_n + cK_X) + w_e[2(w_e + w_n) + cK_X]} \tag{13}$$

$$s_2\,(B_{1e}) = \frac{\frac{1}{4}\gamma_e^2 B_{1e}^2 T_{2e}[2w_n + cK_X]}{\frac{1}{4}\gamma_e^2 B_{1e}^2 T_{2e}(4w_e + 2w_n + cK_X) + w_e[2(w_e + w_n) + cK_X]} \tag{14}$$

and the effective saturation factor s_{eff}:

$$s_{\mathrm{eff}}(B_{1\mathrm{e}}) = \frac{\frac{1}{4}\gamma_{\mathrm{e}}^2 B_{1\mathrm{e}}^2 T_{2\mathrm{e}}[2(w_{\mathrm{e}} + w_{\mathrm{n}}) + cK_{\mathrm{X}}]}{\frac{1}{4}\gamma_{\mathrm{e}}^2 B_{1\mathrm{e}}^2 T_{2\mathrm{e}}(4w_{\mathrm{e}} + 2w_{\mathrm{n}} + cK_{\mathrm{X}}) + w_{\mathrm{e}}[2(w_{\mathrm{e}} + w_{\mathrm{n}}) + cK_{\mathrm{X}}]} \tag{15}$$

Here, w_{e} and w_{n} are electron spin and nuclear spin transition probabilities per unit time related to the observable spin lattice relaxation rates as $w_{\mathrm{e}} = {}^{1}/_{(2 \cdot T_{1e})}$ and $w_{\mathrm{n}} = {}^{1}/_{(2 \cdot T_{1n})}$, c the radical concentration, and K_{X} the Heisenberg exchange constant. The maximum effective saturation factor strongly depends on the interaction between the two EPR resonances, which enables saturation factors close to unity even for systems with hf splitting.[6,13] Similar analytical expressions were given also for a three-line EPR spectrum of unlabeled nitroxides.[15]

Supplementary Note: 1: Characterization of the liquid state DNP probe

The new liquid state DNP probe was characterized by measuring the beam shape and polarization of the MW at the sample position (Supplementary Figure 2) and comparison to electromagnetic field calculations (Supplementary Figure 3). In addition, we also characterized sample heating (Supplementary Figure 4) and the influence of PA concentration on the NMR line shape (Supplementary Figure 5). More details are given in the Methods section and the main text.

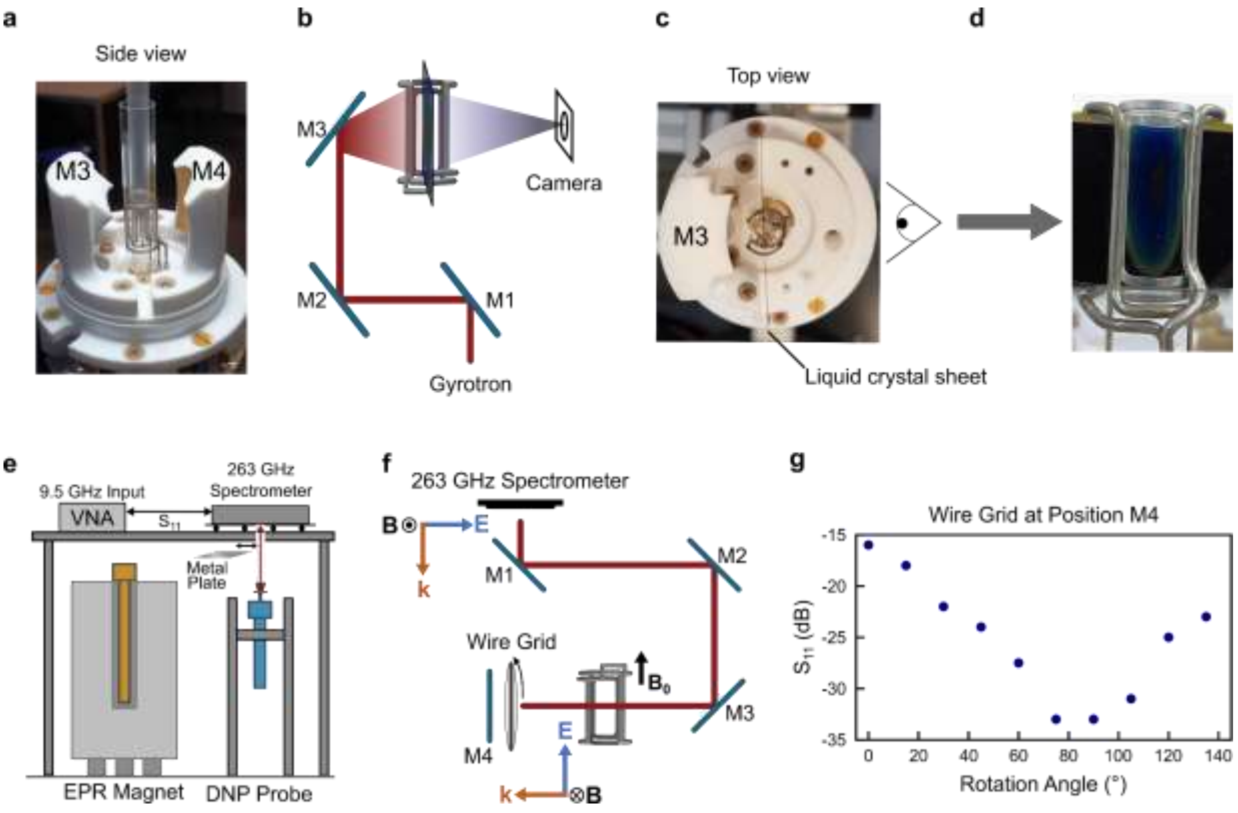

Supplementary Figure 2: (a) Side view of the probe arrangement, including mirrors M3 and M4, the NMR coils, and the NMR coil support quartz tubes. (b) Sketch of the setup for testing the beam alignment, where M4 was replaced by a camera. (c) Top view of mirror M3, the NMR coils and the liquid crystal sheet placed orthogonal to the beam direction. (d) Image of the MW beam spot in the center of the NMR coil. (e) Sketch of the setup used to test the MW beam polarization and MW losses. VNA means vector network analyzer. (f) Sketch of the MW pathway in the probe indicating the incident MW polarization and the polarization at the sample. MW power losses were measured using M4, while for polarization measurements M4 was replaced by a wire grid. Here, **k**, **B**, and **E** are the propagation magnetic field and electric field vectors, respectively. (g) Reflected MW power as a function of the rotation angle of the wire grid. 0° indicates the wire grid being aligned with the **E**-field component of the MW beam, which leads to the maximum of

the S_{11} reading. Figure reproduced with permission from ref.[16] Source data are provided as a Source Data file.[43]

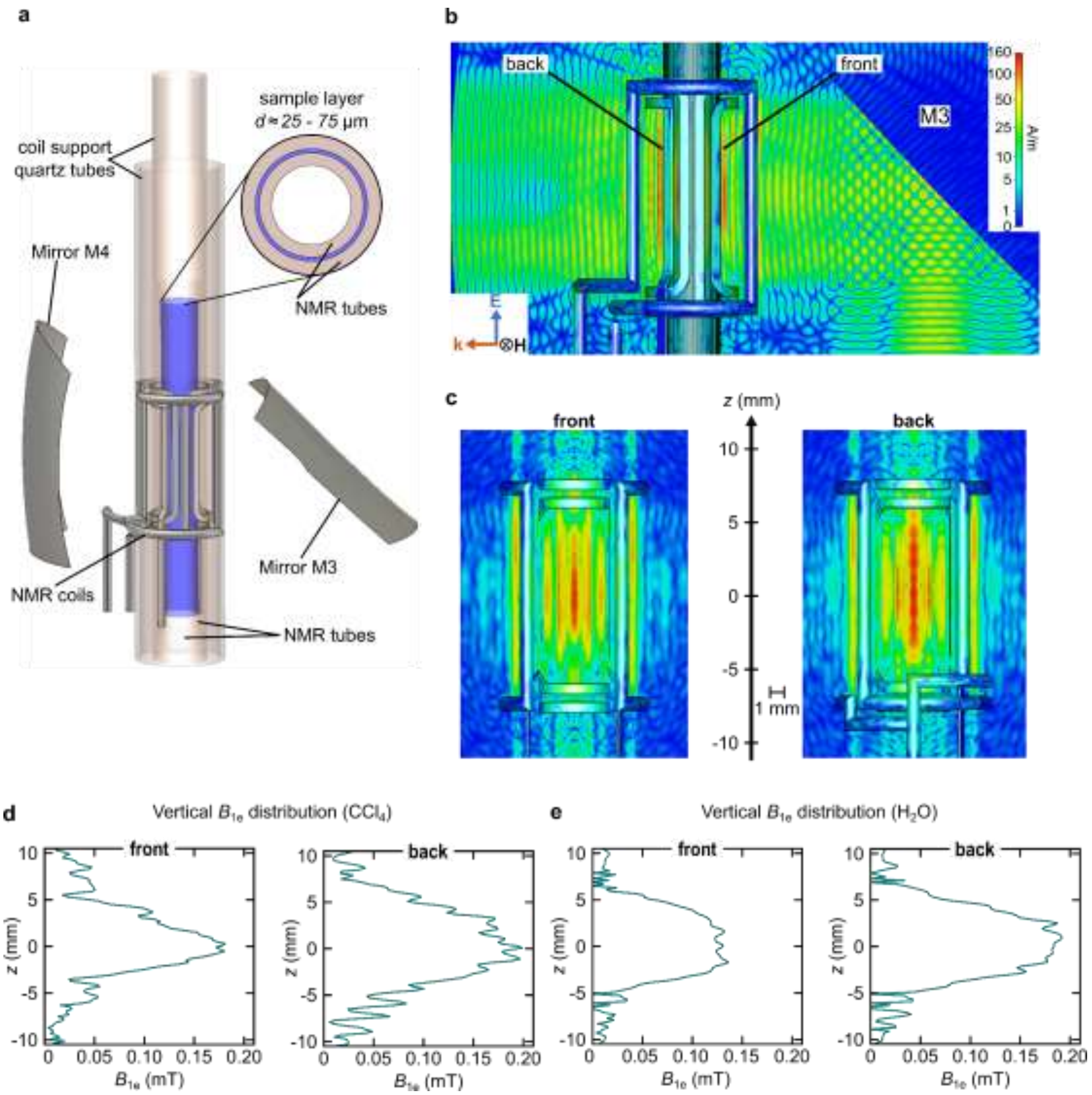

Supplementary Figure 3: (a) Drawing of the sample area. (b) Cross-section of the simulated magnetic **H**-field distribution along the propagation vector **k** in the center of the MW beam with CCl_4 as the sample (Methods). **E** indicates the electric field vector. The absolute field strength is shown for 263.3 GHz and at an oscillation phase of 11°. Power of the excitation beam (Gaussian Beam nearfield source) was 10 W. The NMR support tubes are omitted for clarity. Maxima corresponding to increased energy density are observed at transitions between materials with different ϵ_r. The same scaling was used in panels (b and c). (c) Cross-section of the **H**-field distribution orthogonal to **k** in the sample (CCl_4) at the front and back of the NMR coil window. (d-e) B_{1e} distribution along the z axis with CCl_4 ($d \approx 75$ µm) and H_2O ($d \approx 25$ µm) as the sample. Panels (c-e) show that the MW beam is inhomogeneously distributed in the horizontal and vertical dimension with a clear maximum of about $B_{1e}^{max} \approx 0.2$ mT in the center of the beam. The increased B_{1e} at the back is attributed to reflections from M4 that contribute to an increased standing wave ratio. Panel (e) shows that B_{1e} is only slightly attenuated if H_2O is used instead of CCl_4 but using a 3-fold smaller sample layer. Additionally, the permittivity contrast between H_2O and quartz is larger than for CCl_4, which may further boost the energy density at the sample position. Source data are provided as a Source Data file.[43]

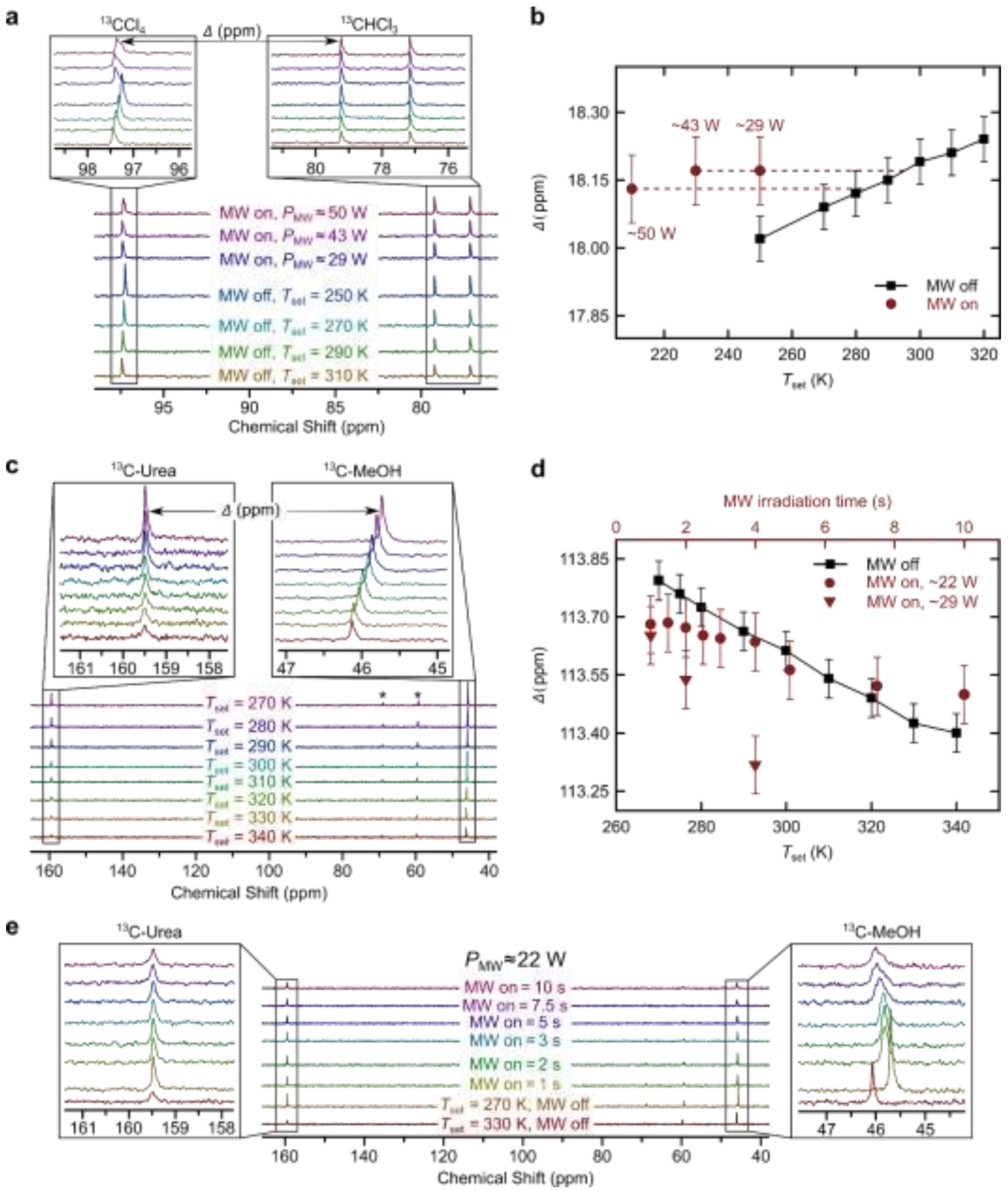

Supplementary Figure 4**:** (a) ^{13}C NMR spectra of a 9/1 CCl_4/$^{13}CHCl_3$ (v/v) mixture (with 10% ^{13}C enrichment CCl_4) using a sample layer of $d \approx 75$ µm as a function of temperature and MW irradiation power. (b) Chemical shift difference of $^{13}CCl_4$ and $^{13}CHCl_3$ as a function of the temperature without MW irradiation (black squares) and upon CW MW irradiation (red diamonds). Lines are a guide to the eye. Error bars in b and d depend on the line shape of the resonance and were assigned to be 0.05 ppm and 0.075 ppm for MW off and MW on measurements, respectively. (c) Temperature dependent ^{13}C NMR spectra of a 250 mM urea-^{13}C and 250 mM methanol-^{13}C in a mixture of water and glycerol 90/10 w/w using a sample layer of $d \approx 25$ µm. (d) Chemical shift difference of urea -$^{13}C_6$ and methanol -$^{13}C_6$ as a function of the temperature without MW irradiation (black squares) and upon gated MW irradiation (red diamonds and spheres). (e) ^{13}C NMR spectra of the same sample as a function of MW irradiation time ($P_{MW} \approx 22$ W). (*) indicates glycerol signals. Figure reproduced with permission from ref.[16] Source data are provided as a Source Data file.[43]

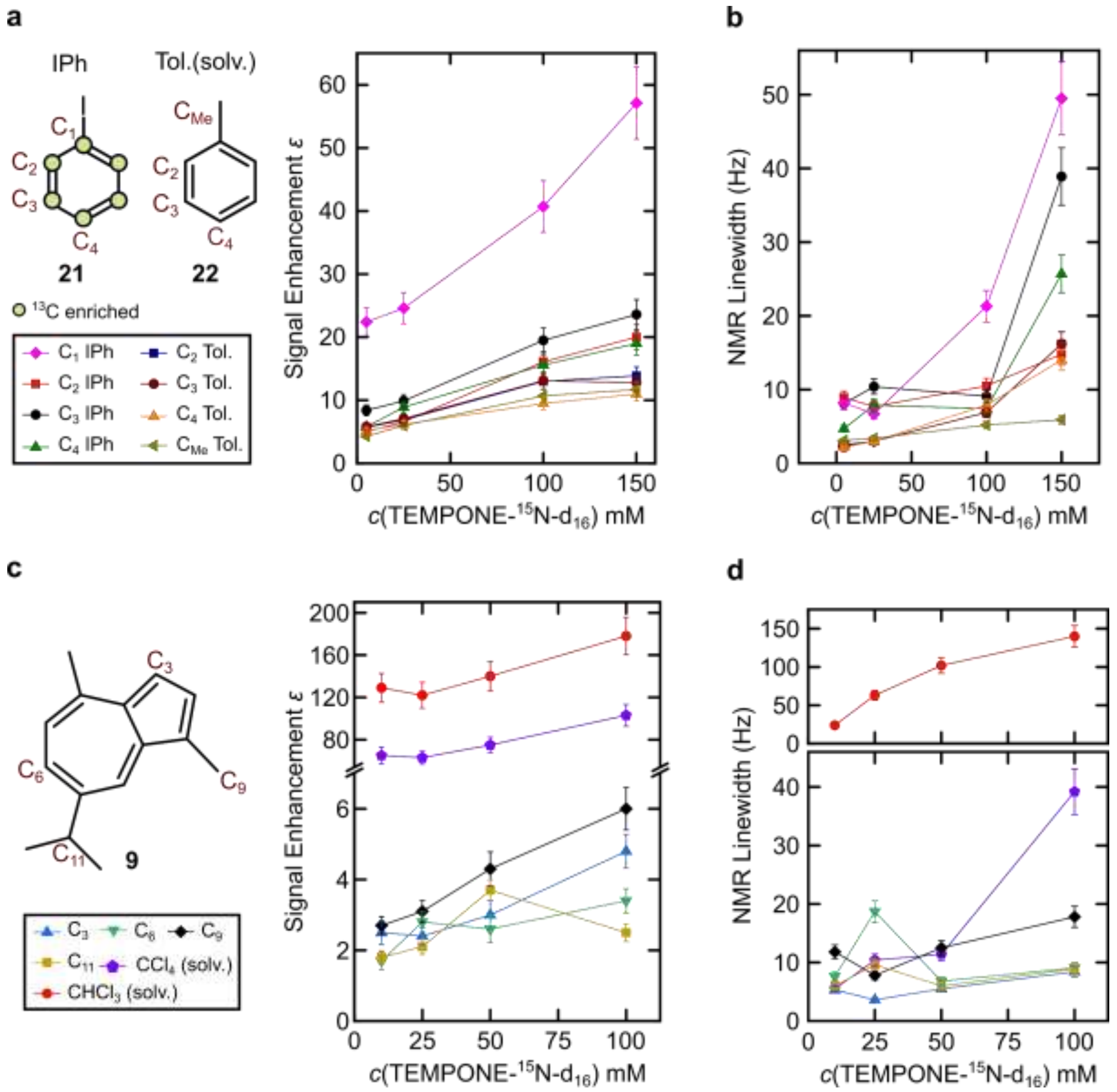

Supplementary Figure 5: (a-b) NMR Signal enhancement and NMR line width dependence of iodobenzene-$^{13}C_6$ **21** ($c \approx 100$ mM) in toluene **22** on the PA concentration (TEMPONE-^{15}N-d_{16}). Line widths of iodobenzene were obtained using the global spectral deconvolution method of the software Mestrenova. (c-d) NMR signal enhancement and line width dependence of guaiazulene **9** ($c \approx 500$ mM) in $CCl_4/CHCl_3$ 9/1 (v/v). All measurements were at an effective sample temperature of ~300 K and $P_{MW} \approx 40$ W. Uncertainty of enhancements and NMR linewidths is estimated to be 10%. Source data are provided as a Source Data file.[43]

Supplementary Note: 2: Characterization of the frequency agile gyrotron

Liquid-state DNP experiments require stable MW irradiation (several days) on resonance with a narrow (3 - 10 MHz) EPR line. Moreover, the MW frequency has to be tuned in a range of about 500 MHz (Fig.1c, main text). Therefore, we purchased a custom-designed gyrotron produced by Communications & Power Industries (CPI) and supplied by Bruker BioSpin, which is equipped with a 4.8 T cryogen-free magnet and produces MW radiation with frequencies around 263 GHz. The instrument allows to adjust the MW frequency in a range of $\Delta\nu \approx 200$ MHz at an output power of ~10 – 50 W and in a range of $\Delta\nu \approx 500$ MHz at an output power of ~10 W. Frequency and power were adjusted by changing the different gyrotron parameters, that are highlighted in Supplementary Figure 6a. Frequency and power were monitored with a calibrated water load and a frequency meter (Virginia Diodes Inc.), which was connected at the end of the corrugated waveguide. Supplementary Figure 6b representatively reports the output power as well as the MW frequency for a frequency range of $\Delta\nu \approx 200$ MHz at an output power of ~40 W. The sweep range can be further extended to $\Delta\nu \approx 500$ MHz at an output power of ~10 W by varying the main magnetic field of the gyrotron. We monitored the frequency stability with the same setup by overnight measurements and found a frequency drift of $\lesssim 0.6$ MHz, independent on the output power (see Supplementary Table 1).

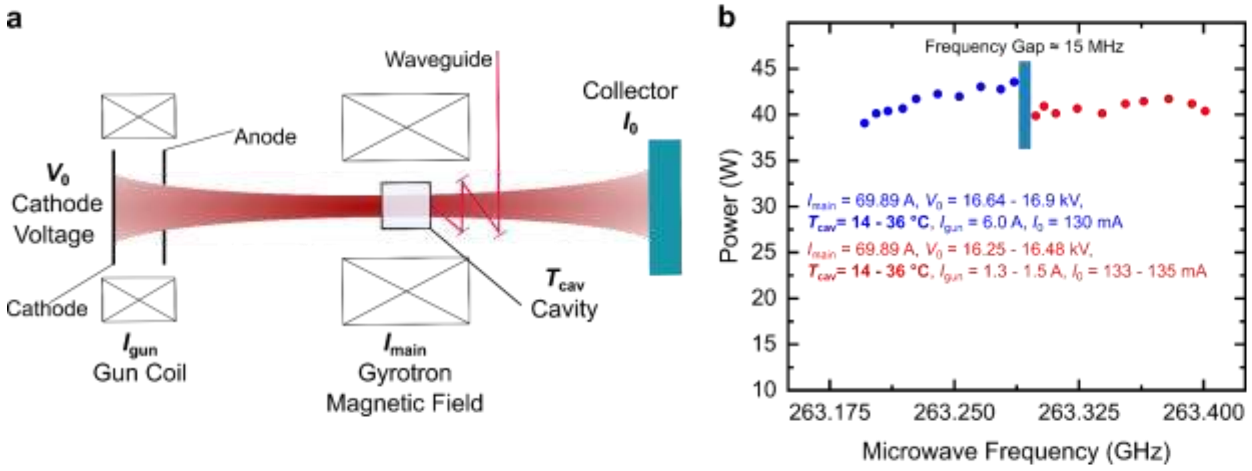

Supplementary Figure 6: (a) Schematic of the gyrotron vacuum tube[17] highlighting the different adjustable parameters such as the cathode voltage V_0, the gun current I_{gun}, the cavity temperature T_{cav}, and the collector current I_0. (b) Frequency sweep at an output power set to 40 W upon variation of the cavity temperature and the other parameters indicated in (a). Figure reproduced with permission from ref.[16] Source data are provided as a Source Data file.[43]

Supplementary Table 1: Frequency drift and power monitoring of the gyrotron at different power levels. The measurement started at t_0 and ended at t_1 ($\Delta t = t_1 - t_0$ and $\Delta \nu$ = deviation of microwave frequency over the indicated time period). (*) Measurement performed by Communications & Power Industries (CPI).

power (W)	MW frequency (t_0, GHz)	MW frequency (t_1, GHz)	$\Delta \nu$ (MHz)	Δt (h)
41	263.4014	263.4019	0.5	12.5
12	263.2946	263.2952	0.6	16
62	263.232	263.232	-	336*

Supplementary Note 3: 1D DNP NMR spectra in non-polar and polar solvents

In the following, we report all individual ^{13}C and ^{19}F NMR spectra with DNP (MW on) and without DNP (MW off) illustrated in Fig. 1g and Fig. 2 (main text). The experimental error is estimated to be 10 – 15 % and mainly originates from the temperature gradient during DNP experiments and the signal-to-noise ratio (SNR) of the Boltzmann measurement. Sample temperature was ~300 K and, if not noted otherwise, the solvent was CCl_4. NMR experimental parameters are given in the Methods. Data shown in Supplementary Figures 7 and 9 were reported in the PhD thesis of one of the authors.[16]

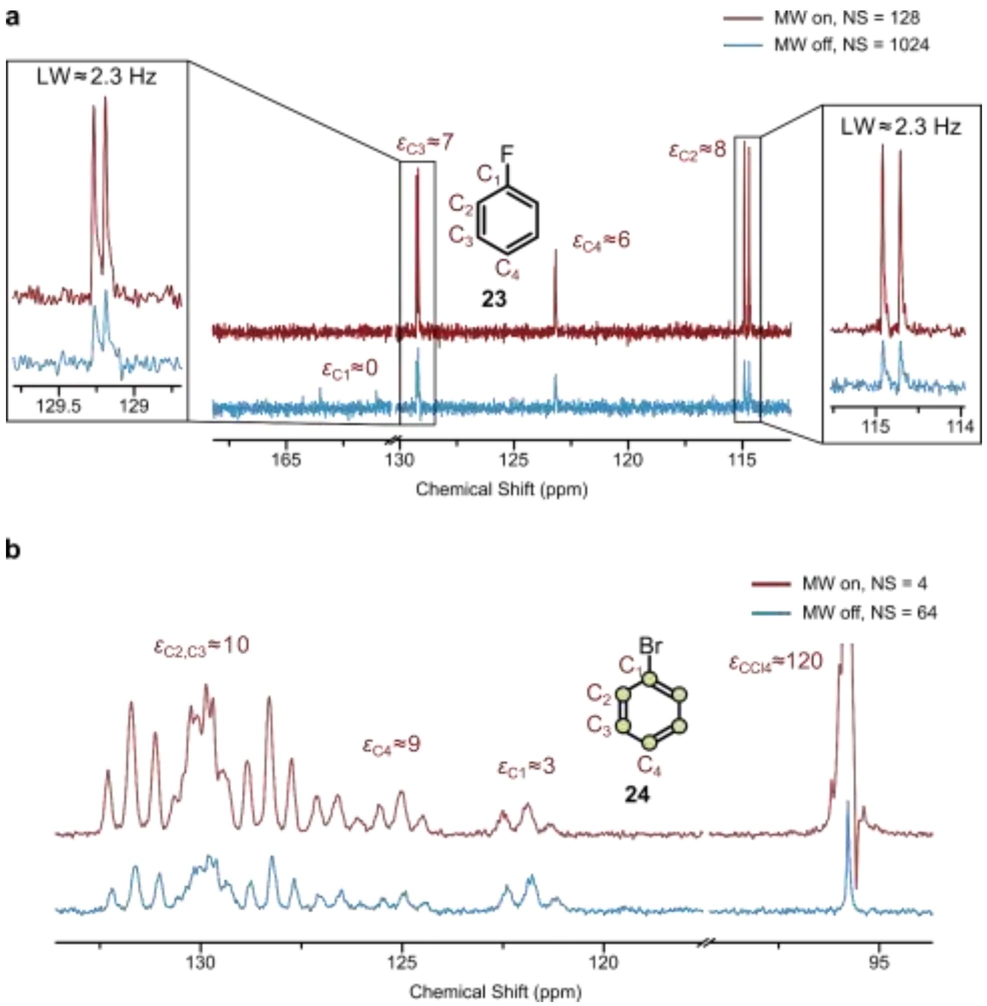

Supplementary Figure 7: (a) ^{1}H decoupled ^{13}C NMR DNP spectra of fluorobenzene **23** with zoom-in-views on the C_3 position and the C_2. (b) ^{1}H decoupled ,^{13}C NMR DNP spectra of bromobenzene-$^{13}C_6$ **24** in CCl_4. Spectra were scaled to the same SNR. Source data are provided as a Source Data file.[43]

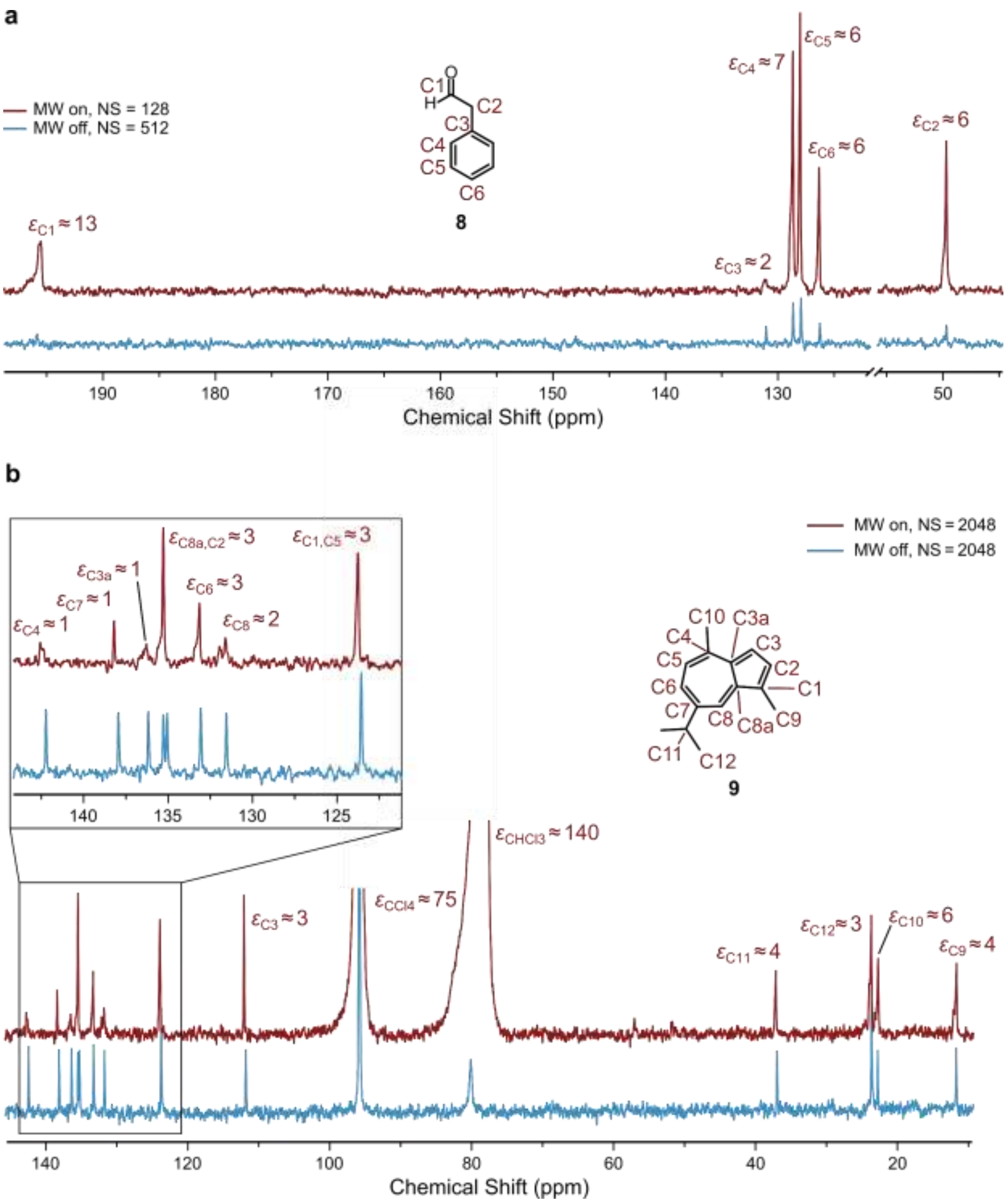

Supplementary Figure 8: (a) ^{1}H decoupled ^{13}C NMR DNP spectra of phenylacetaldehyde **8** ($c \approx$ 1 M, c(PA) $\approx$ 50 mM in CCl_4). Spectra were scaled to the same SNR. (b) ^{13}C NMR DNP spectra of 500 mM guaiazulene **9** in $CHCl_3$ (c(TEMPONE$-^{15}$N-d$_{16}$) $\approx$ 50 mM). DNP spectrum was acquired using ~35 W MW power and enhancements of guaiazulene were scaled to 40 W. Source data are provided as a Source Data file. [43]

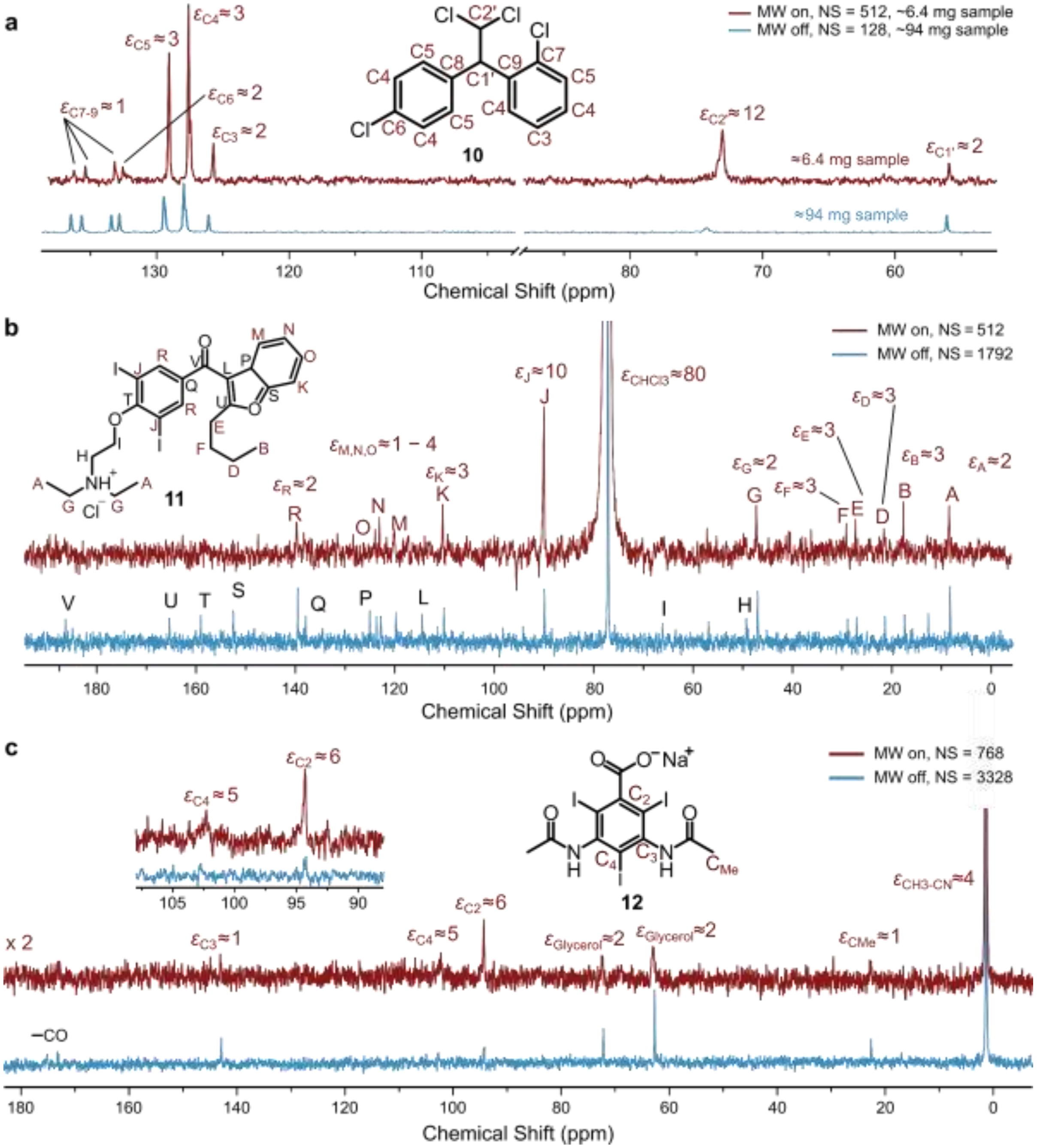

Supplementary Figure 9: (a) ^{1}H decoupled ^{13}C NMR DNP spectra of mitotane **10** ($c \approx 500$ mM, $c(\mathrm{PA}) \approx 25$ mM in CCl_4). The thermal spectrum was acquired using a ~12 times larger sample volume. MW on and off spectra were divided by their number of scans and volumetric ratio. (b) ^{1}H decoupled ^{13}C NMR DNP spectra of amiodarone hydrochloride **11** in $CHCl_3$. Spectra were divided by their number of scans. (c) ^{1}H decoupled ^{13}C NMR DNP spectra of Na diatrizoate **12** in H_2O/glycerol 9/1 ($c(\mathrm{TEMPONE{-}^{15}N\text{-}d_{16}}) \approx 25$ mM). Experimental parameters: $P_{\mathrm{MW}} \approx 22 - 29$ W, MW irradiation time = 4 – 6 s and RD = 30 s. Spectra were scaled to the SNR and the DNP spectrum was multiplied by 2 for better visualization. Source data are provided as a Source Data file.[43]

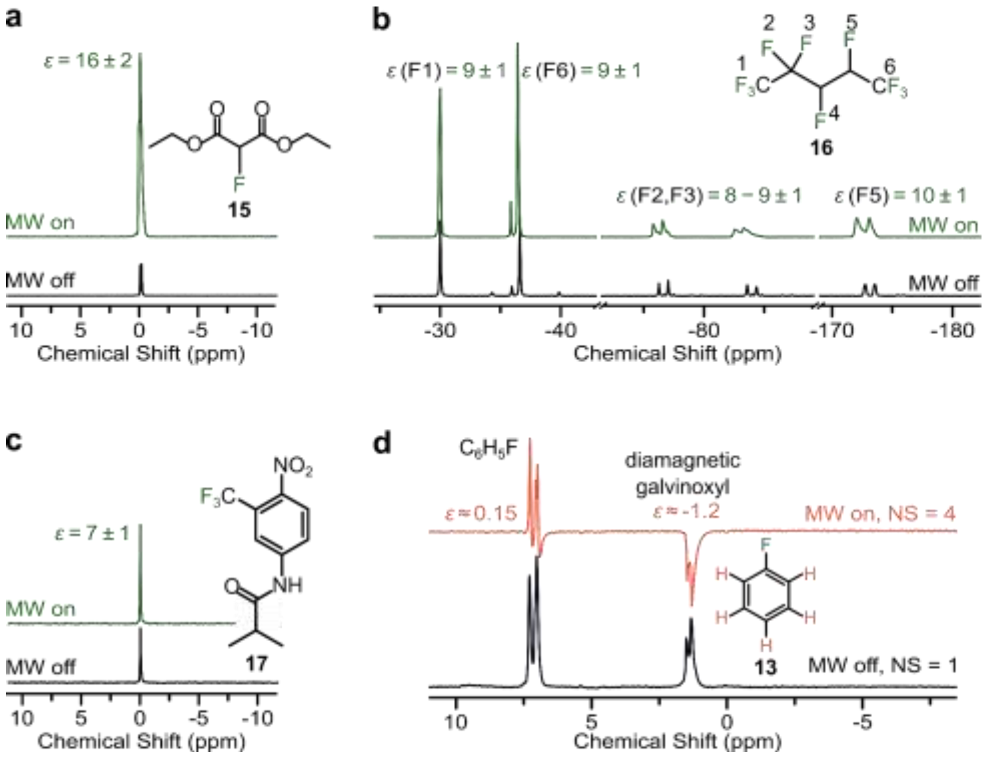

Supplementary Figure 10: (a) ^{19}F NMR DNP of diethyl fluoromalonate **15** ($c \approx 500$ mM). (b) ^{19}F NMR DNP of decafluoropentane **16** ($c \approx 500$ mM). (c) ^{19}F NMR DNP of flutamide **17** ($c \approx 10$ mM) using ~330 mM DMSO to increase the solubility of the target molecule. Spectra were scaled to the same SNR. Spectra were either centered at 0 ppm (a,c) or are reported unreferenced (b), due to the lack of an internal standard. (d) ^{1}H NMR DNP spectra of fluorobenzene **13** ($c \approx 500$ mM, $c(PA) \approx 25$ mM). The intense second signal is assigned to the methyl groups of diamagnetic galvinoxyl. All measurements were performed with 25 mM of galvinoxyl as a PA and CCl_4 as a solvent. Detected ^{19}F and ^{1}H are highlighted in green and orange, respectively. Source data are provided as a Source Data file.[43]

Supplementary Note 4: DNP enhanced 2D ^{13}C-^{13}C correlation NMR experiments

Suplementary Table 2: DNP enhancements of 2D ^{13}C -TOCSY spectra of ethyl acetoacetate-1,2,3,4-$^{13}C_4$ and 3-hydroxybut-2-enoate-1,2,3,4-$^{13}C_4$ (c(PA) ≈ 25 mM). Dashes mark correlation peaks with insufficient SNR for enhancement evaluation. 1D enhancements were evaluated based on the ^{13}C NMR spectrum with inverse-gated decoupling on ^{1}H (as in Fig.3a). Table prepared with data extracted from spectra provided as source data files.[43]

Site 1 (indirect dimension)	Site 2 (direct dimension)	Enhancement	1D enhancement for site 1
C3	C3	0.5	0.5
	C1	-	
	C2	-	
	C4	1.5	
C1	C3	-	0.8
	C1	0.6	
	C2	0.6	
	C4	-	
C2	C3	9.0	11.5
	C1	13	
	C2	11	
	C4	12	
C4	C3	9	9.7
	C1	9	
	C2	9	
	C4	8	
C7	C7	0.9	0.6
	C5	0.7	
	C6	-	
	C8	-	
C5	C7	0.6	0.5
	C5	0.8	
	C6	0.6	
	C8	-	
C6	C7	7	7
	C5	6	
	C6	7	
	C8	-	
C8	C7	10	8
	C5	-	
	C6	13	
	C8	7	

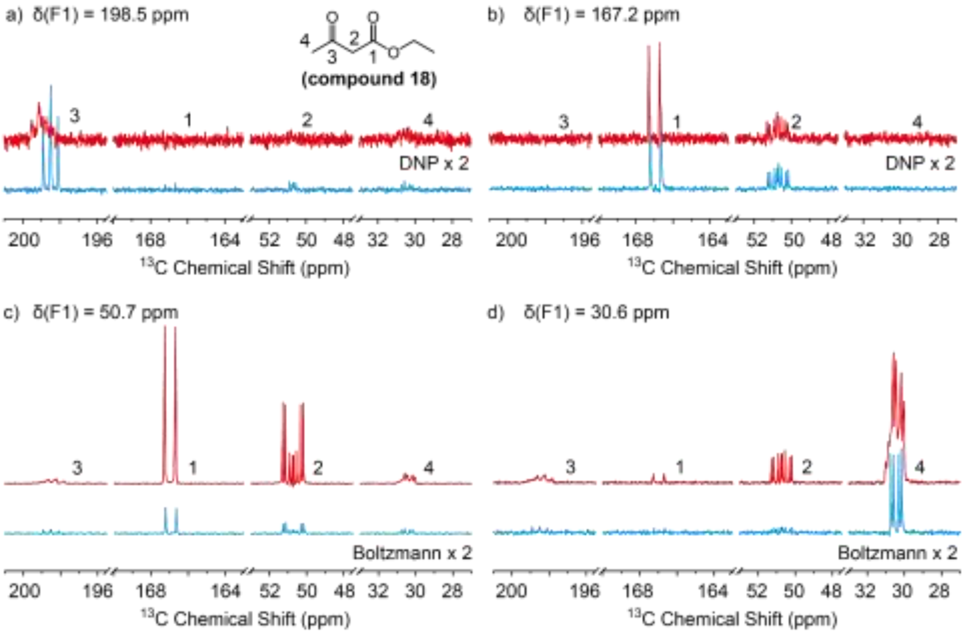

Supplementary Figure 11: 1D slices of the 2D ^{13}C-TOCSY spectra of ethyl acetoacetate-1,2,3,4-$^{13}C_4$ with 9.4 ms isotropic mixing measured under DNP (red traces) and Boltzmann (blue traces) conditions at δ(F1) = 198.5 ppm (C3) (a), δ(F1) = 167.2 ppm (C1) (b), δ(F1) = 50.7 ppm (C2) (c), δ(F1) = 30.6 ppm (C4) (d) in the F1 dimension (see Fig. 3a for the notation). Source data provided as a source data file.[43]

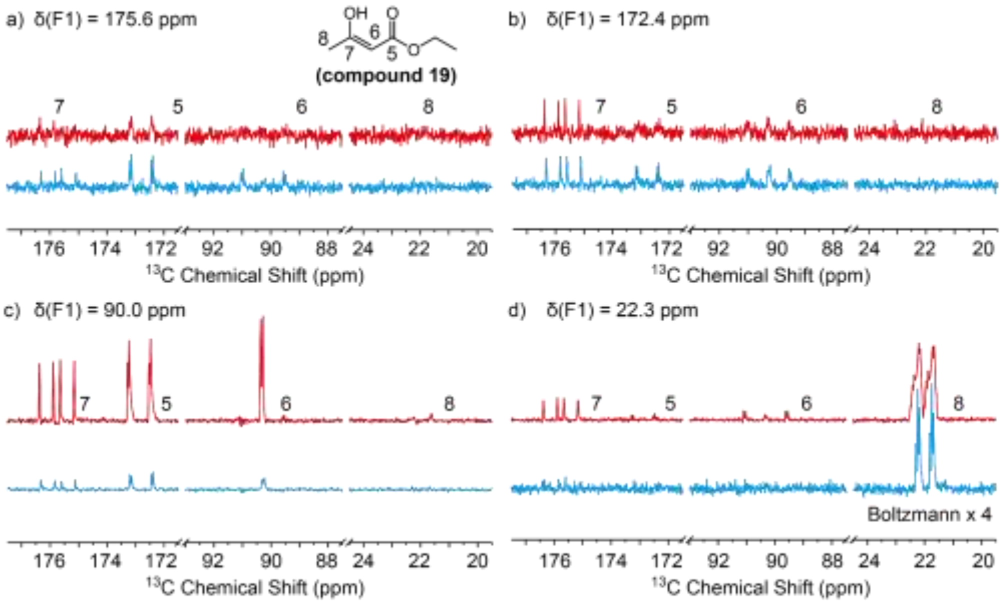

Supplementary Figure 12: 1D slices of the 2D ^{13}C-TOCSY spectra of ethyl 3-hydroxybut-2-enoate-1,2,3,4-$^{13}C_4$ with 9.4 ms isotropic mixing measured under DNP (red traces) and Boltzmann (blue traces) conditions at δ(F1) = 175.6 ppm (C7) (a), δ(F1) = 172.4 ppm (C5) (b), δ(F1) = 90.0 ppm (C6) (c), δ(F1) = 22.3 ppm (C8) (d) in the F1 dimension. Source data provided as a source data file.[43]

Supplementary Table 3: Enhancements of the ^{13}C-^{13}C correlation peaks in the 2D DNP-^{13}C-TOCSY experiment of 500 mM iodobenzene-$^{13}C_6$ (c(PA) ≈ 25 mM) in cyclohexane (Supplementary Figures 13-14). 1D enhancements were evaluated based on ^{13}C NMR spectrum with inverse-gated decoupling on ^1H. Table prepared with data extracted from spectra provided as source data files.[43]

Site 1 (indirect dimension)	Site 2 (direct dimension)	Enhancement	1D enhancement for site 1
C1	C1	26	27
	C2	28	
	C3	29	
	C4	28	
C2	C1	9	10
	C2	14	
	C3	14	
	C4	10	
C3	C1	14	12
	C2	14	
	C3	13	
	C4	12	
C4	C1	-	13
	C2	14	
	C3	15	
	C4	14	

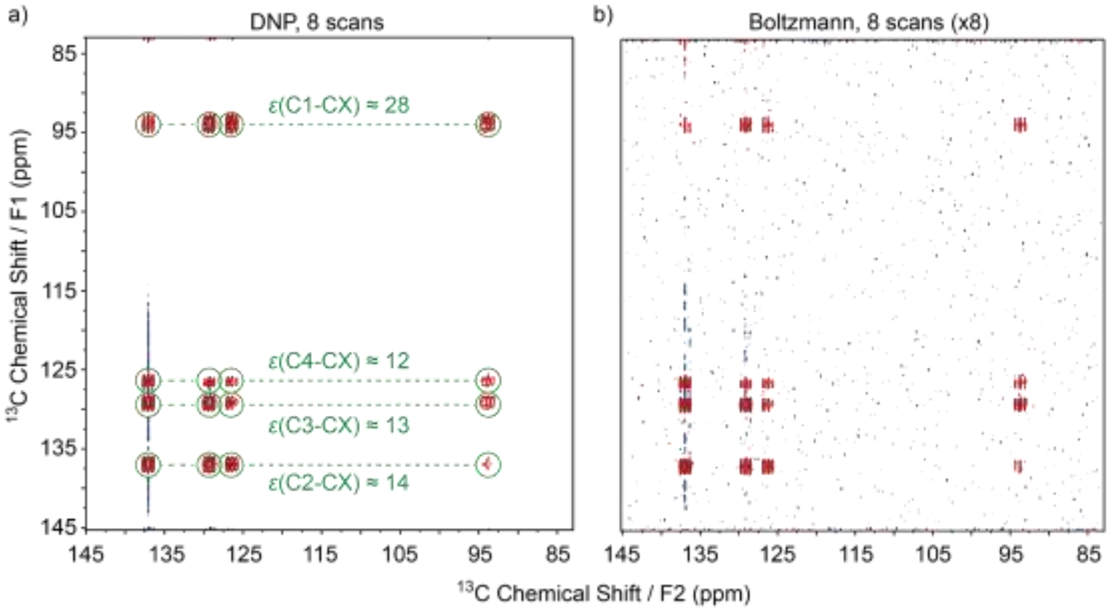

Supplementary Figure 13 (a, b) 2D ^{13}C-TOCSY spectra of 500 mM iodobenzene-$^{13}C_6$ **21** in cyclohexane under DNP (a) and Boltzmann (b). For isotropic mixing, 34.5 ms of DIPSI-2 spin-lock[18] was implemented. Intensities in spectrum in (b) is multiplied by 8 for clarity. Red and blue contours indicate positive and negative values, respectively. Source data are provided as a Source Data file.[43]

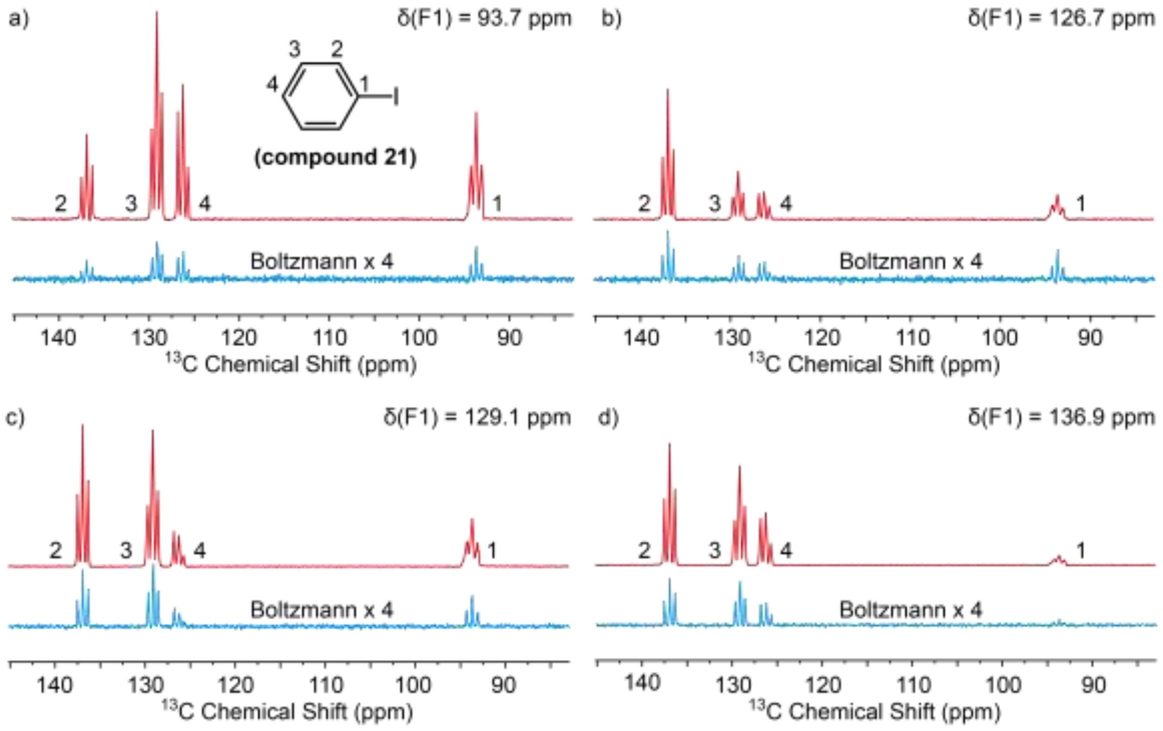

Supplementary Figure 14: (a-d) 1D slices of the 2D ^{13}C-TOCSY spectra of idobenzene-$^{13}C_6$ **21** measured under DNP (red traces) and Boltzmann (blue traces) conditions at δ(F1) = 93.7 ppm (C1) (a), δ(F1) = 126.7 ppm (C4) (b), δ(F1) = 129.1 ppm (C3) (c), and δ(F1) = 136.9 ppm (C2) (d) in the F1 dimension. Source data are provided as a Source Data file.[43]

Supplementary Table 4: 2D enhancements of the correlation peaks of ^{13}C-DNP-INADEQUATE of 1-fluoro-4-iodobenzene (1.5 M in cyclohexane, with 25 mM TEMPONE-^{15}N-d_{16}). Table prepared with data extracted from spectra provided as source data files.[43]

Double-quantum label (F1 dimension)	2D enhancement from 1D slices	1D enhancement	Average of 1D enhancements, $(\varepsilon_1 + \varepsilon_2)/2$
C1-C2	20	ε(C1) ≈ 25<br>ε(C2) ≈ 15	20
C2-C3	13	ε(C2) ≈ 15<br>ε(C3) ≈ 20	17.5
C3-C4	8	ε(C3) ≈ 20<br>ε(C4) ≈ 0	10

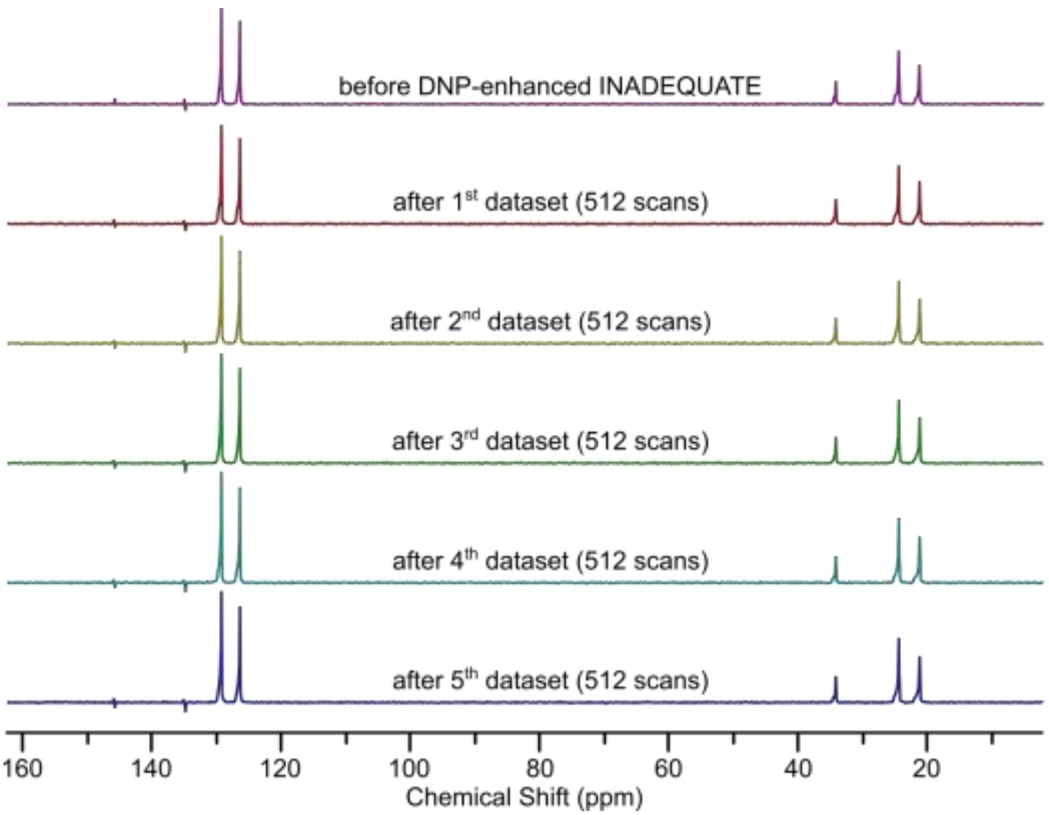

Supplementary Figure 15: 1D ^{13}C NMR control spectra under MW irradiation of neat (6.4 M) p-cymene (nat. abun.) doped with 100 mM TEMPONE-^{15}N-d_{16} (64 scans) recorded before and during the DNP-enhanced ^{13}C-INADEQUATE (see main text Fig.4). Each INADEQUATE dataset took 28 hours. Source data are provided as a Source Data file.[43]

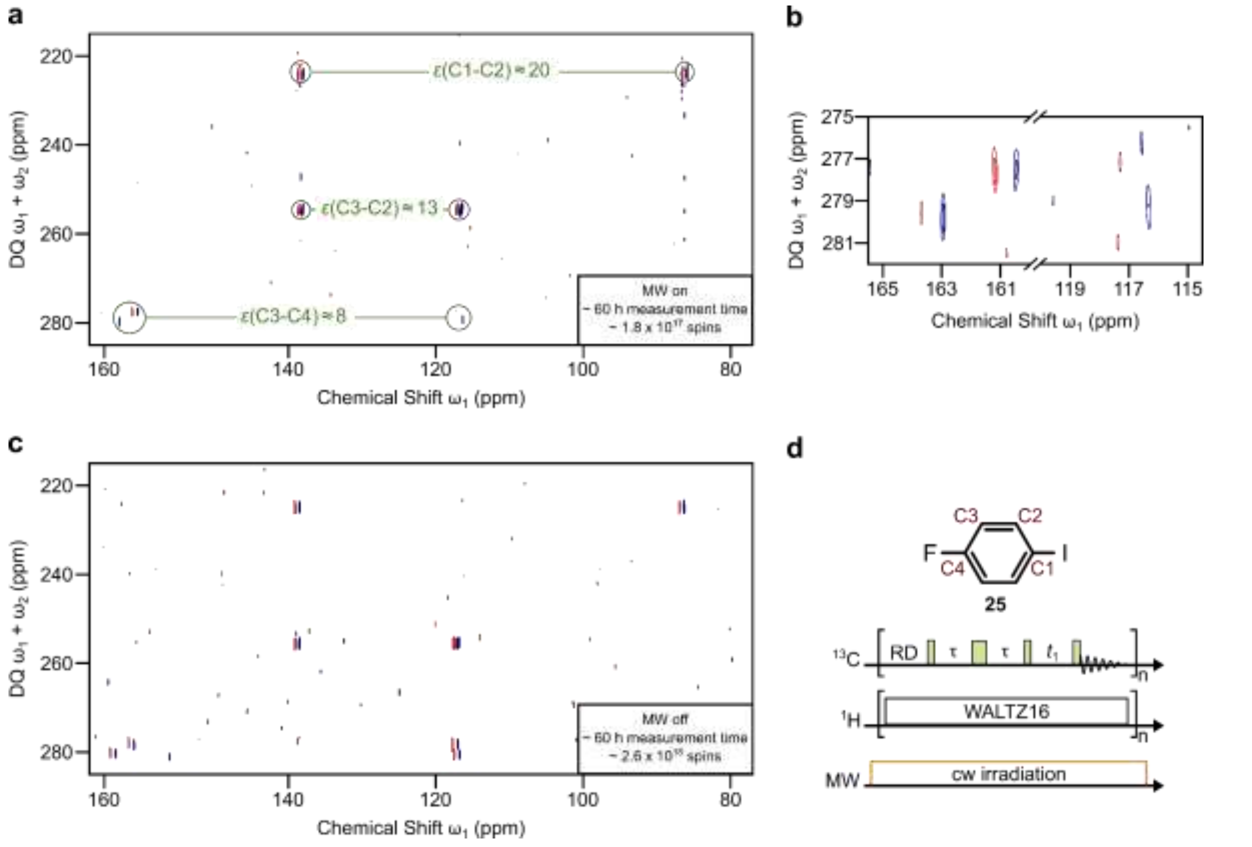

Supplementary Figure 16: (a) DNP-enhanced 2D ^{13}C-INADEQUATE of 1.5 M 1-fluoro-4-iodobenzene **25** (nat. abun.) in cyclohexane doped with 25 mM TEMPONE-^{15}N-d_{16} with 2048 × 128 points and 288 scans. Red and blue contours indicate positive and negative values, respectively. (b) Zoom-in view on the C3+C4 correlation signal. (c) Boltzmann 2D ^{13}C-INADEQUATE spectrum of natural-abundant 1-fluoro-4-iodobenzene with the same PA and target molecule concentration as used for the DNP experiment but ~15 times larger sample volume i.e. a full 5 mm NMR tube. (d) Pulse sequence for DNP-enhanced 2D ^{13}C-INADEQUATE. Green narrow and wide rectangles represent 90° and 180° pulses. Grey and orange bars represent heteronuclear decoupling (WALTZ-16) and MW irradiation, respectively. τ is set to $1/4J_{CC}$ = 55 Hz and t_1 is incremented; RD is the recycle delay. Source data are provided as a Source Data file.[43]

Supplementary Discussion 1: Electron spin relaxation times at 263 GHz, room temperature, and experimental saturation factor

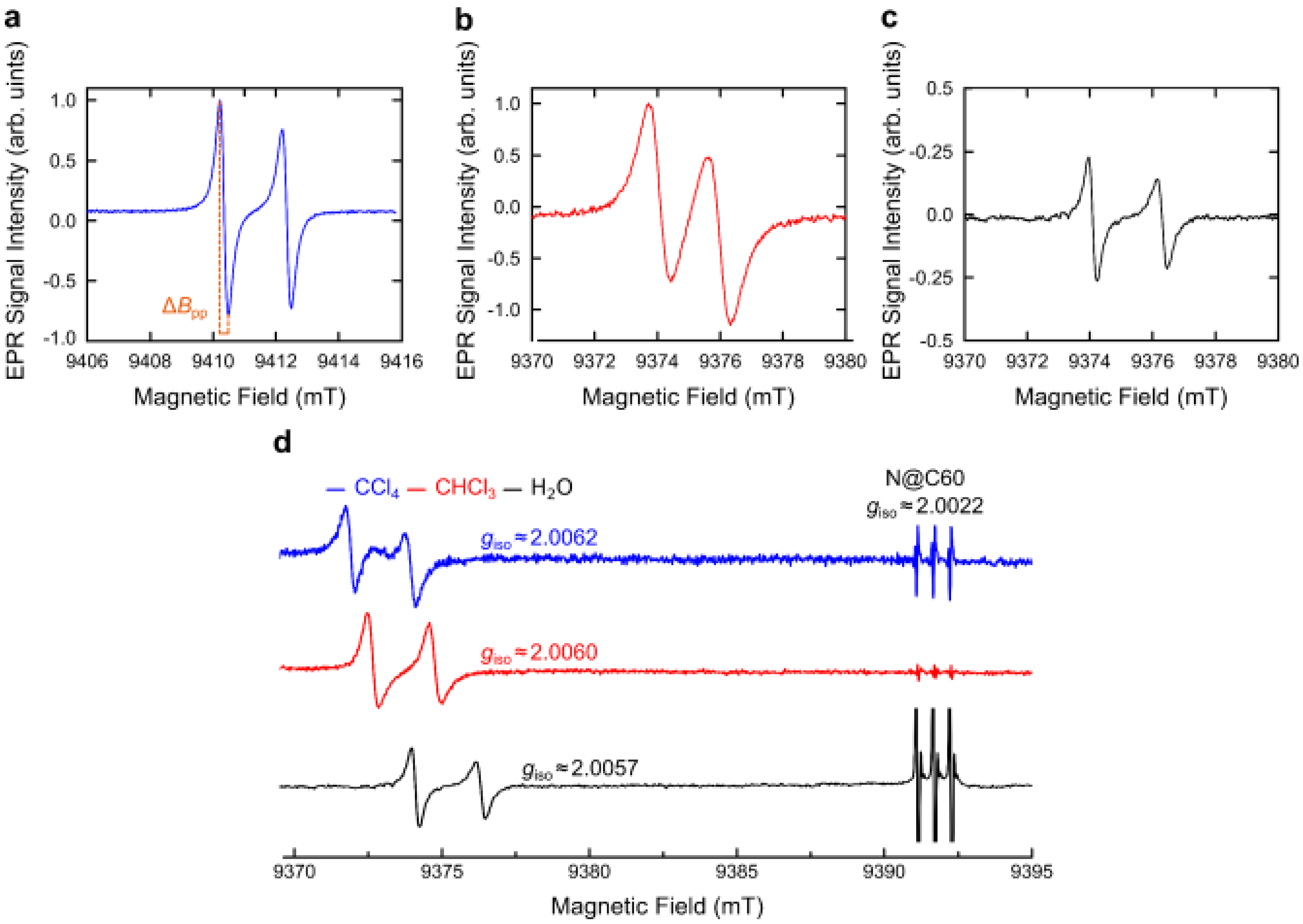

Supplementary Figure 17: (a-c) CW EPR measurements at 263 GHz, T = 295 K of TEMPONE-^{15}N-d$_{16}$ in CCl_4 ($c \approx 5 - 15$ mM), $CHCl_3$ ($c \approx 20$ mM), and H_2O ($c \approx 10$ mM). Results are summarized in Supplementary Table 5. (a) sample #2, (b) sample #5, and (c) sample #7. Numbering of the sample is according to Supplementary Table 5. The full spectrum of (c) is depicted in in panel d (black line). Experimental parameters: modulation frequency (MF) = 100 kHz, modulation amplitude (MA) = 0.01 – 0.15 mT, receiver gain (RG) = 39 dB, $P \approx 0.5 - 5$ mW, NS = 1 – 5, 1024 – 2700 points. (d) 263 GHz CW-EPR spectra of TEMPONE-^{15}N-d$_{16}$ in CCl_4 (blue, $c \approx 2$ mM), $CHCl_3$ (red, $c \approx 2$ mM), and H_2O (black, $c \approx 10$ mM), corresponding to sample #4, #6, and #7 in Supplementary Table 5, using N@C_{60} as a g factor reference, which was calibrated in-house to a carbon fiber.[19] Data shown was initially reported in the PhD thesis of one the authors.[16] Source data are provided as a Source Data file.[43]

Knowledge of the longitudinal (T_{1e}) and transversal (T_{2e}) electron spin relaxation times at 263 GHz enables the calculation of the saturation factor (eq. 15) as a function of the microwave field strength B_{1e}. This is crucial to rationalize and predict DNP enhancements based on eq. 2. Thus, we determined T_{1e} and T_{2e} at 263 GHz of TEMPONE-^{15}N-d_{16} at room temperature for most representative solvents such as CCl_4, $CHCl_3$, and H_2O. Details of the 263 GHz EPR experiments are given in Methods. Deoxygenated CW EPR spectra of the samples #1 – #3 were collected in a non-resonant probe, while CW EPR of samples #4 – 7 were collected either in a cylindrical TE_{012} resonator or in a single mode (TE_{011}) resonator (Methods). Pulsed EPR experiments were performed in a TE_{012} resonator.

T_{2e} Relaxation times

The transversal electron spin relaxation time T_{2e} is obtained from the first derivative of the 263 GHz CW EPR spectrum by measuring the peak-to-peak separation (ΔB_{pp}) [20,21]

$$T_{2e} = \frac{2}{\sqrt{3}\Delta B_{pp}\gamma_e} \tag{16}$$

Here, ΔB_{pp} is in Tesla and γ_e in $\frac{\mathrm{Hz \cdot rad}}{\mathrm{T}}$. Supplementary Figure 17a-c shows representative CW EPR spectra of three different samples of TEMPONE-^{15}N-d_{16} in CCl_4, $CHCl_3$, and H_2O. Degassing was

Supplementary Table 5: Peak-to-peak separation ΔB_{pp} and T_{2e} values obtained from the low field line of the CW EPR spectrum (this work) of TEMPONE-^{15}N-d_{16}. (*) Measurements were performed in presence of N@C_{60} for g factor referencing (see Supplementary Figure 17). Experimental errors are $\leq 5 - 10$ %. Table prepared with data extracted from spectra provided as source data files.[43]

	Sample #1	Sample #2	Sample #3	Sample #4*	Sample #5	Sample #6*	Sample #7*	ref. [22]	ref. [23]
solvent	CCl_4	CCl_4	CCl_4	CCl_4	$CHCl_3$	$CHCl_3$	H_2O	H_2O	H_2O
degassed	yes	yes	yes	no	no	no	no	–	–
c (PA) (mM)	5 – 15	5 – 15	5 – 15	2	20	2	10	50	3
ΔB_{pp} (mT)	0.31	0.26	0.26	0.33	0.68	0.41	0.27	–	–
T_{2e} (ns)	21	25	25	20	10	16	24	28	15 – 25
g_{iso}				2.0062		2.0060	2.0057		

omitted for the CW EPR measurements in $CHCl_3$ and H_2O. The experimental results of all three solvents are summarized in Supplementary Table 5. The experiments show that despite the solvent variation and the different oxygen content, the T_{2e} values are all in the range of 10 – 30 ns at radical concentrations $c \approx 2 - 30$ mM. The line widths are much larger than the contribution from unresolved deuteron couplings (about 0.016 mT) that dominates at X-band.[24] This means line widths are dominated by some combination of incomplete motional average of anisotropy, which is concentration independent, collisions with O_2 and nitroxide-nitroxide collisions, that are concentration dependent (see for instance sample # 5).

T_{1e} Relaxation times

T_{1e} was measured at T = 295 K with 263 GHz pulsed EPR using inversion recovery (IR)[25] experiments. Experiments were performed on TEMPONE-^{15}N-d_{16} in CCl_4, because this serves as a model system for DNP measurements at 9.4 T. Supplementary Figure 18a and b show the experiments at 263 GHz on two duplicate samples with $c(\text{TEMPONE}-^{15}\text{N-d}_{16}) \approx 10$ mM in CCl_4. Details on sample preparation and experimental parameters are given in the Methods. Due to the difficult sealing of the thin capillaries, oxygen penetrated over time into the sample. This is evidenced by a slow decreasing of T_{1e} over time (Supplementary Figure 18b and c). Therefore, reproducibility of the results was verified by measuring a total of six samples replicates. Corresponding sample labels and T_{1e} values are reported in Supplementary Table 6. Fitting of the time trace to a bi-exponential function[6] $y = A\exp\left(\frac{-\tau}{T_{1e}}\right) + B\exp\left(\frac{-\tau}{T_b}\right)$, yields T_{1e}. The second exponential T_b accounts for effects that may arise from the MW pulse at small τ values.

Supplementary Table 6: T_{1e} of six replicate samples of ~10 mM TEMPONE-^{15}N-d_{16} in CCl_4. All experiments were performed under comparable experimental conditions except for the precise content of oxygen. (*) $c(\text{TEMPONE}-^{15}\text{N-d}_{16}) \approx 10$ mM. Spread of T_{1e} values reflects the different oxygen content, which cannot be controlled. Data shown was initially reported in the PhD thesis of one the authors.[16] Table prepared with data extracted from spectra provided as source data files.[43]

Sample	#8	#9	#10	#11	#12	#13	Ave.
T_{1e} (ns)	380	300	270	225*	240	320	290

We find that the obtained T_{1e} values at 263 GHz are in the same range as our previous results at 3.4 T.[26] A theoretical model for T_{1e} of nitroxides in liquid suggested that T_{1e} should be constant for magnetic fields larger than ~ 1 T.[27] Application of this model to our results in CCl_4, using the only free parameter $\tau_C \approx 4$ ps, allowed us to simulate the trend in the experimental data within the experimental error (Supplementary Figure 19) using:

$$\frac{1}{T_{1e}} = \frac{1}{T_{1e}^{SR}} + \frac{1}{T_{1e}^{A}} + \frac{1}{T_{1e}^{g}} + \frac{1}{T_{1e}^{therm}} \tag{17}$$

Here, SR refers to spin rotation, A to hyperfine anisotropy, g to *g* anisotropy, and therm to a thermal contribution to T_{1e}. More details on the model are provided in ref.[27], other parameters are given in the figure caption. As compared to our previous study,[26] where τ_C was determined from CW EPR line shape analysis to $\tau_C \approx 8$ ps, we note that the simulated τ_C is underestimated likely due to the large error on T_{1e} at high fields. Therefore, these values seem to provide a robust estimate for the saturation factor based on eq. 15 as presented in Supplementary Figure 20. Ref.[23] predicted $T_{1e} \approx 850$ ns at 9.4 T in H_2O, using the model from the aforementioned study[27] that predicts a constant T_{1e} at magnetic fields larger than 1.2 T. For water, $\tau_C = 9$ ps was used.[27,28]

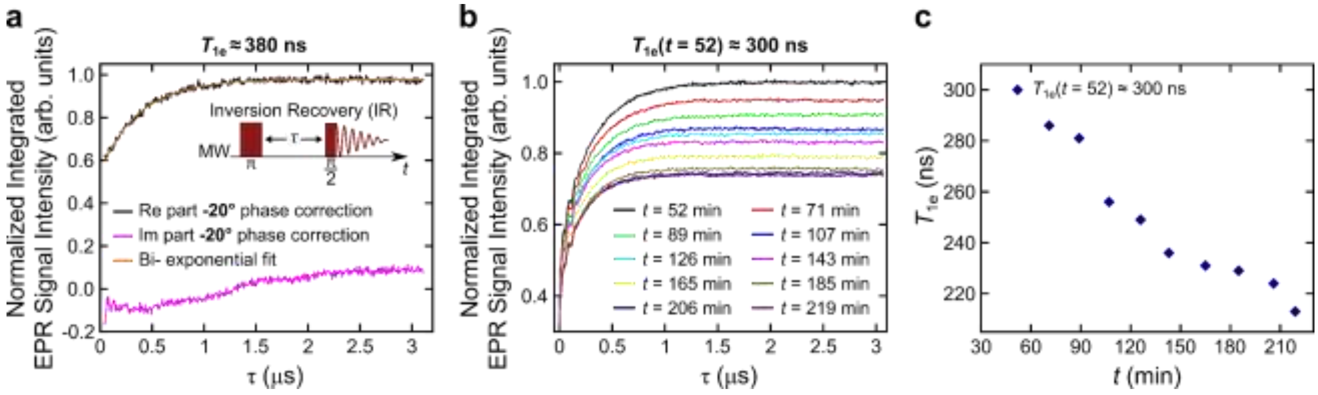

Supplementary Figure 18 (a) Pulse sequence for inversion recovery experiments is displayed in the inset. Rel and imaginary part of the recovery traces for sample #8. Experimental parameters: $\nu = 264.2417$ GHz, $B_0 = 9.4311$ T, $P(\text{EPR}) \approx 50$ mW, $t_p\left(\frac{\pi}{2}\right) = 100$ ns, $\tau = 4$ ns (time increment), 512 points, dead time after detection pulse = 146 ns. (b) IR experiments of sample #9 at different time points t after removing the sample from N_2 atmosphere. (c) T_{1e} values obtained at different delays t from the first measurement. Figure reproduced with permission from ref.[16] Source data are provided as a Source Data file.[43]

Lastly, we used the experimental electron spin relaxation parameters and the saturation factor to calculate the average microwave field strength B_{1e} at the sample position. The saturation factor was calculated using the experimental signal enhancement of CCl_4 and literature data for f and ξ.[9] Insertion of these experimental data into eq. 15 leads to a $B_{1e} \approx 0.045 - 0.065$ mT at $P_{MW} \approx 40$ W (Supplementary Figure 20). More details are given in the methods section.

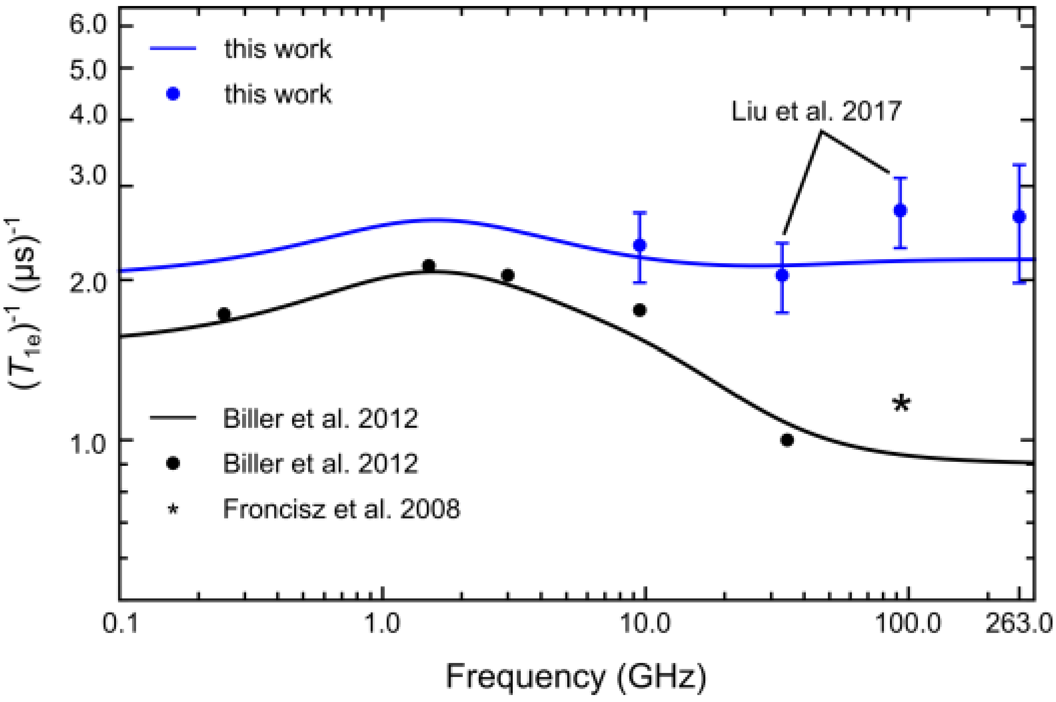

Supplementary Figure 19: Experimental (circles) and simulation (lines) of T_{1e} of TEMPONE-^{15}N-d$_{16}$ in CCl_4 (blue, our work ref[26]) and of TEMPONE in H_2O (black[23,27,29]) as a function of the resonance frequency. Experimental error for measurements at 9.5 – 93 GHz was estimated to be ~15 %, while for 263 GHz an uncertainty of ~25 % was assumed (sample #8 is depicted here). Note, the uncertainty of the data point at 94 GHz may be slightly larger, than reported in ref.[26], possibly due to challenging sample preparation under O_2 exclusion. The simulation was performed with protocol and parameters introduced in ref.[27]: g = [2.0092, 2.0061, 2.0022], A = [15.4, 17.7, 99.7] MHz, I (^{15}N) = ½, $C_{therm} = 7.0 \times 10^{16}$, τ_{therm} = 100 ps and ω_{ref} = 9.5 GHz. More information on the model can be found in the literature ref.[27,28] A was adjusted for the simulation in CCl_4 to be $A = [29, 29, 127]$ MHz[30]) with the rotational correlation time being the sole free parameter ($\tau_C = 3.7$ ps). Figure reproduced with permission from ref.[16] Source data provided as a source data file.[43]

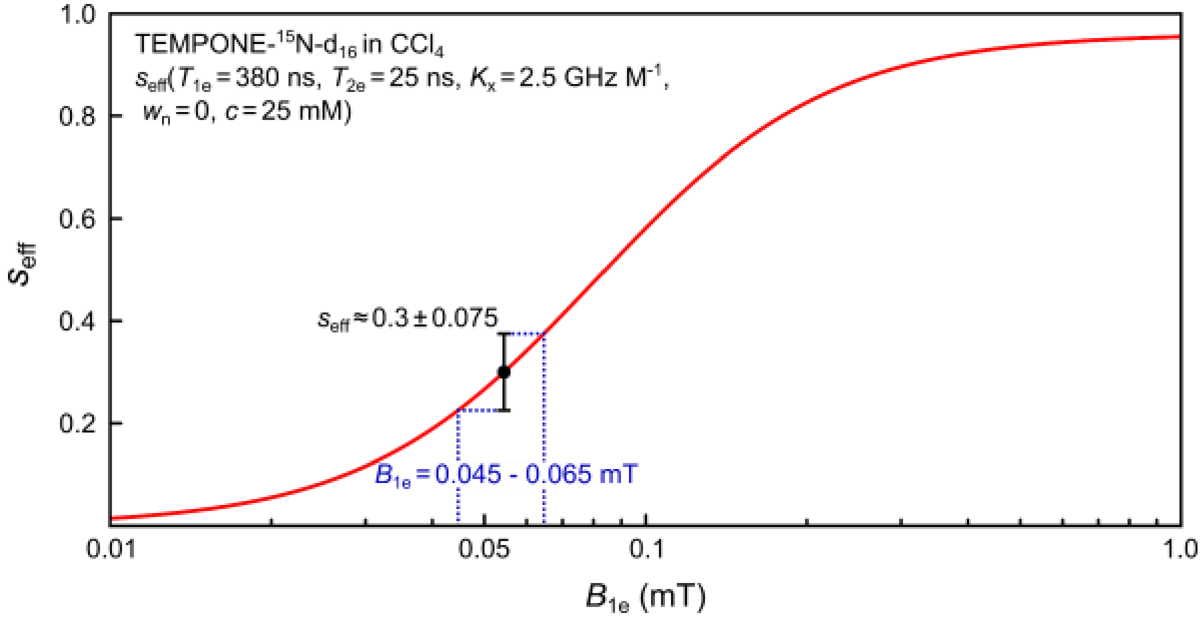

Supplementary Figure 20: (a) Calculation of s_{eff} as a function of B_{1e} using eq.15 and the experimentally observed relaxation parameters of TEMPONE-^{15}N-d_{16} in CCl_4, as given in the figure legend. As the deoxygenation of DNP samples is experimentally less challenging and therefore more efficient than for T_{1e} and T_{2e} experiments, maximum values for T_{1e} and T_{2e} are expected to be representative for the relaxation behavior of DNP samples (i.e. experimental values of samples #2 and #8 were used). We used K_X from the literature.[26] From the DNP signal enhancement, s_{eff} = 0.35 ± 0.075 was calculated and is marked in the diagram. The uncertainty of s was estimated to be ~25%. Comparison of this value with the calculated curves enabled us estimate an average microwave field strength of $B_{1e} \approx 0.045 - 0.065$ mT ($P_{MW} \approx$ 40-50 W) at the sample position.

Supplementary Discussion 2: Mechanistic interpretation of the aromatic ^{13}C enhancements at 9.4 Tesla

In order to rationalize NMR signal enhancements obtained on aromatic carbons, we investigated the magnetic field dependence of the coupling factor ξ (^{13}C) for three representative target molecules, i.e. benzene, fluorobenzene and iodobenzene. Values of ξ (^{13}C) were extracted according to eq. 2, using independent measurements of the Overhauser parameters s and f, as listed in Supplementary Table 7 for 9.4 T and Supplementary Table 8 for 1.2 T. At both external fields, 1.2 T and 9.4 T, ξ is negative: this suggests that the dipolar contribution to cross-relaxation has decayed, while the scalar part, modulated by molecular collisions, remains an active cross-relaxation pathway and enables signal enhancements at high fields, as expressed by eq. 8. This is observed for a large variety of small molecules, which display positive enhancements at 9.4 T (Fig. 2, main text). Supplementary Table 7 shows that coupling factors for protonated aromatic carbons C2 – C4 at 9.4 T are all similar in value ($|\xi| \approx 1 - 1.5$ %), while the iodinated carbon of iodobenzene has a larger coupling factor of $|\xi| \approx 4\,\%$. Although these values appear small, they give rise to one to two orders of magnitude enhancements, due to the large ratio $|\gamma_e|/\gamma_{13C} \approx 2600$. Moreover, the coupling factor decreases only by a factor of 3 - 4 between 1 and 9 T.

Supplementary Table 7: Overhauser parameters at 9.4 Tesla for fluorobenzene-$^{13}C_6$, iodobenzene-$^{13}C_6$, and benzene-$^{13}C_6$ doped with 10 – 25 mM TEMPONE-^{15}N-d_{16}. (*) Saturation factor estimated as described in the Methods. Estimated error in ξ is 25 %. For T_{1n}^0 of fluorobenzene-$^{13}C_6$ two measurements were performed and the average value is reported here. In cases where C_2-C_4 were resolved in the NMR spectrum, the average of these three positions is reported. Table prepared with data extracted from spectra provided as source data files.[43]

Carbon Position	ε	s*	f	T_{1n} (s)	T_{1n}^0 (s)	ξ
			fluorobenzene-$^{13}C_6$			
C_1	0	0.3	0.9	5.4	55.5	0.0014
C_2-C_4	6 – 8	0.3	0.83	4.1	23.7	- 0.01
			iodobenzene-$^{13}C_6$			
C_1	25	0.3	0.85	5.2	35.5	- 0.036
C_2-C_4	9	0.3	0.7	4.0	12.5	- 0.015
			benzene-$^{13}C_6$			
C_1	7	0.3	0.64	9.9	27.5	- 0.01

We rationalized the coupling factors in two steps, first by computing A_{iso} values with DFT and then simulating the field dependence of ξ within the frame work of eq. 6, 7 and 9. DFT was used to compute the optimized geometry of the target/radical pair (see Supplementary Figure 21 for representative structures) and calculating the isotropic component of the hf coupling A_{iso} (see Supplementary Figure 22b). The halogenated benzenes form a halogen bond-like complex with the radical with the halogen atom pointing to the oxygen atom of the nitroxide. Interestingly, fluorobenzene is the only one where the halogen (F) faces away from the NO group of the nitroxide (see Supplementary Figure 21) and indeed it shows no enhancement at the ipso (C1) position, while the C_{ipso} position of iodobenzene shows the largest ε and A_{iso} (see Supplementary Figure 22a). The experimental signal enhancement for the halogenated positions overall correlate with the calculated A_{iso}, however precise details will require further investigations. Notably, the measured signal enhancement for all CH groups of the halogenated benzenes are similar in size, a behavior that is also found for the hf coupling of these positions (Supplementary Figure 22b).

Supplementary Table 8**:** Overhauser parameters at 1.2 Tesla for fluorobenzene-$^{13}C_6$, iodobenzene-$^{13}C_6$, and benzene-$^{13}C_6$ doped with ~10 mM TEMPONE-^{15}N-d_{16}. For determination of T_{1n}, two measurements were performed and both results are reported. In all cases, T_{1n} measurements suffered from poor SNR, which led to an increased uncertainty of at least 25 % for T_{1n} and T_{1n}^0. (*) T_{1n} determination of C_1 was not feasible due to poor SNR. Therefore, we assumed a leakage factor of 0.5 – 1.0 to calculate ξ of C_1. The error for ξ is up to 25 %. Table prepared with data extracted from spectra provided as source data files.[43]

Carbon Position	ε	s	f	T_{1n} (s)	$T_{Build\text{-}up}$ (s)	T_{1n}^0 (s)	ξ
			fluorobenzene-$^{13}C_6$				
C_1	-3.8	0.09	0.5 – 1.0*	-	-	-	0.02 – 0.04
C_2-C_4	5	0.09	0.45 – 0.65	8 – 13	3 – 4.5	24.1	-0.038 – (-0.026)
			iodobenzene-$^{13}C_6$				
C_1	18.5	0.35	0.5 – 1.0*	-	-	-	-0.04 – (-0.02)
C_2-C_4	21	0.35	0.17 – 0.26	6.4 – 7.2	6.4	8.7	-0.11 – (-0.07)
			benzene-$^{13}C_6$				
C_1	15	0.28	0.57	12.44	7.1	29.3	-0.033

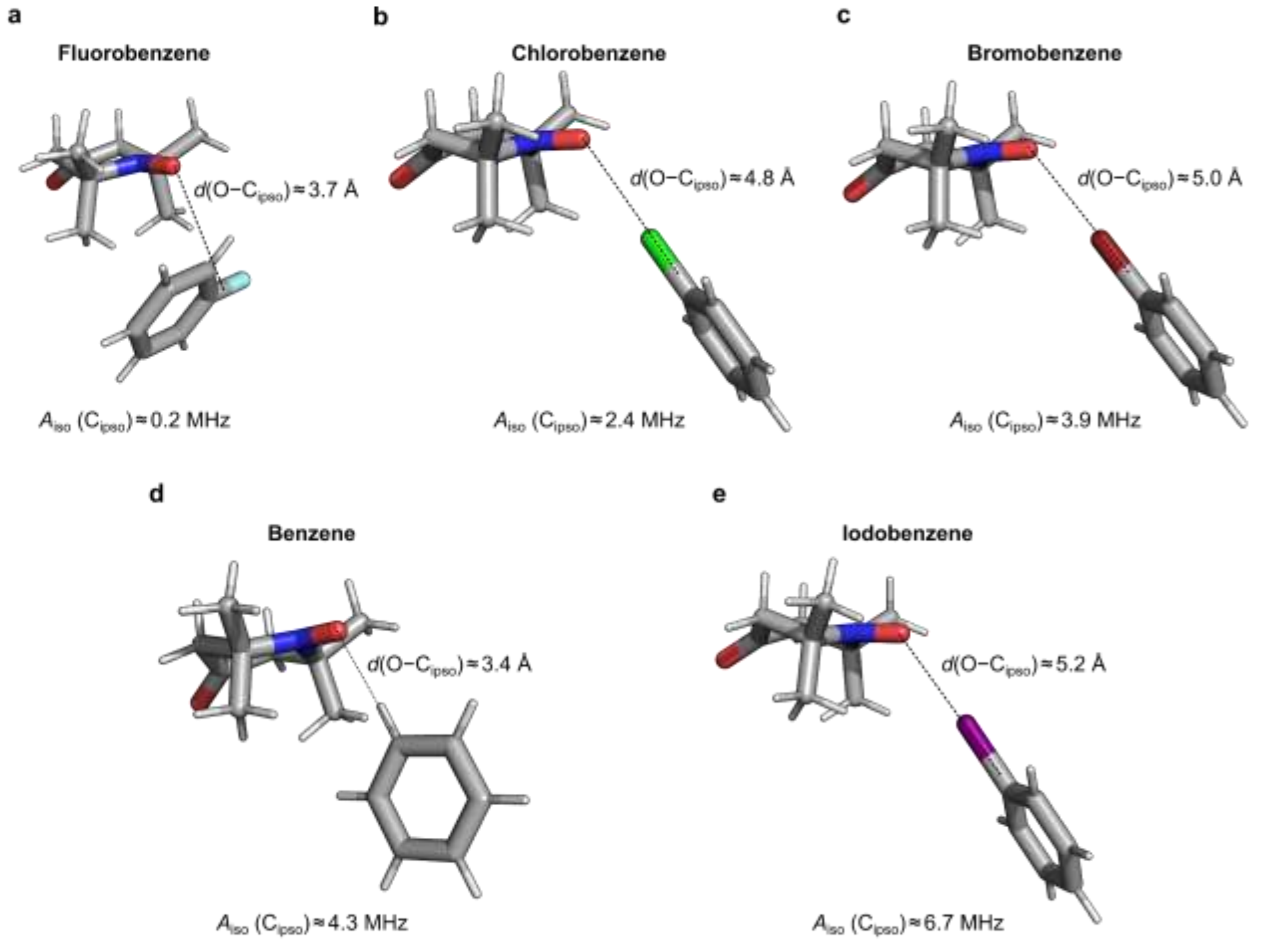

Supplementary Figure 21: Optimized complex structures of TEMPONE and benzene and its mono-halogenated (F, Cl, Br, I) derivatives. Calculations were performed using Orca 5.0.2 (and 5.0.4).[31] Geometries of benzene and mono-halogenated benzenes with TEMPONE were optimized using the B3LYP functional and the def2-TZVPP basis set. D3BJ was employed as a dispersion correction and we also used resolution-of-the-identity and the chains-of-spheres approximation (RIJCOSX with def2/J auxiliary basis set). Implicit solvation model C-PCM (CCl_4)[32,33] and very tight conversion criteria for SCF (VERYTIGHTSCF) and the optimization procedure (TIGHTOPT) were used. Final geometries were obtained from a starting geometry, where the halogen was facing the NO group of the nitroxides. Isotropic hf couplings to C were calculated using the EPR-III basis set (H, C, N, F, and O),[31] IGLO-III for Cl,[34] DKH-TZVPP for Br,[35,36] and SARC-DKH-TZVPP for I.[37] Source data are provided as a Source Data file.[43]

Previous extensive studies on molecules such as CCl_4 and $CHCl_3$[9,12,26,38] indicated that it is critical to have sub-picoseconds modulations of the hf coupling during the lifetime of the target/radical complex (tens of ps). Thus, we simulated the coupling factor of the CH nuclei of halogenated benzenes as a function of the magnetic field with eq.6 and found that it requires short correlation times of $\tau_{sc} \approx 0.3 - 1$ ps (Supplementary Figure 23). To avoid an over- parameterization, we define ranges for the different fit parameters that are based on the literature.[9,13,39,40] The simulation of ξ as a function of B_0 further suggests that also at larger magnetic fields (14.1 T and 18.8 T) appreciable signal enhancements

of $\varepsilon \approx$ 30 should be accessible, a hypothesis that was already confirmed for some model systems.[41] Furthermore, our data show significant signal enhancements for different target molecules (see Fig.2 main text and Supplementary Figures 7-9), specifically on groups that are prone to form hydrogen bonds[39] or halogen bonds (-CCl_3 group and -CI group) with the nitroxide. This is also in agreement with previous reports, which suggested that more acidic protons lead to larger signal enhancements of the attached ^{13}C nucleus[39] and our observations of large signal enhancements for small halogenated solvent molecules.[9,26] On the contrary, groups that are not inclined to interact with the NO group of the nitroxide, such as -CF and -CO, show a suppression of the DNP enhancements (Supplementary Figures 7-9 and ref.[26,42]).

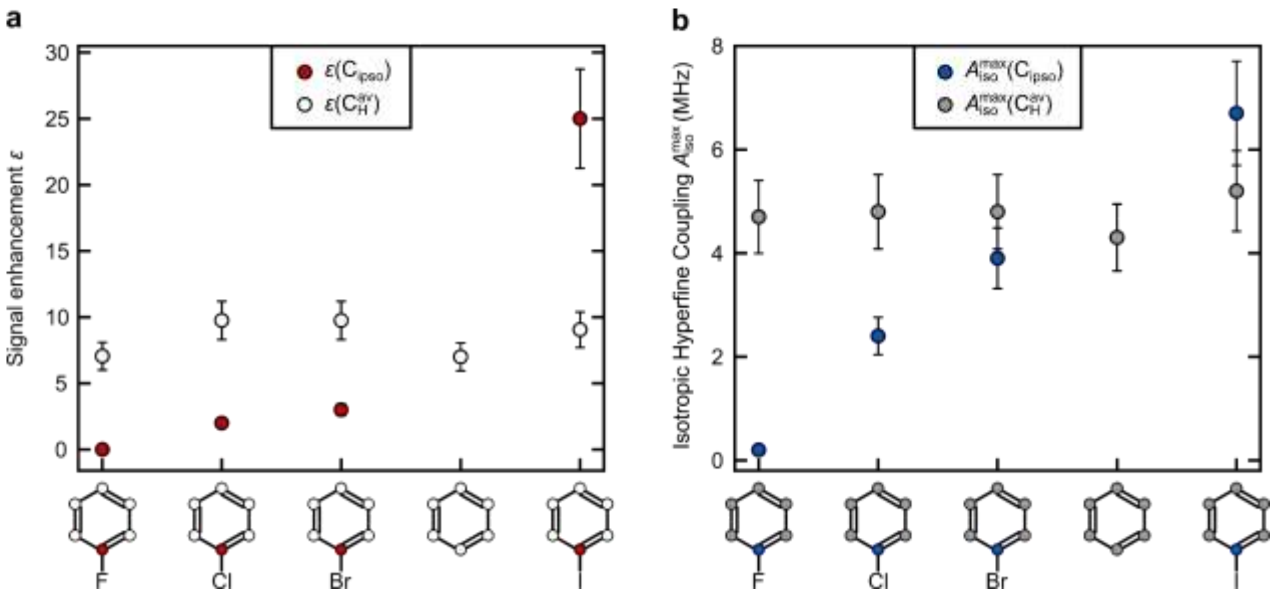

Supplementary Figure 22: (a) Experimental signal enhancement of C_{ipso} and C_H, i.e. ^{13}C nuclei bound to a halogen and hydrogen, respectively. (b) Maximum isotropic hf coupling A_{iso}^{max} of different carbon positions calculated from the representative structures depicted in Supplementary Figure 21. Maximum isotropic hf couplings of hydrogenated positions were calculated from optimized structures. Notably, for C_H, we plot the average enhancement and hf coupling of the five positions, respectively. Experimental and simulations uncertainties are estimated to be ~15 %. Source data are provided as a Source Data file.[43]

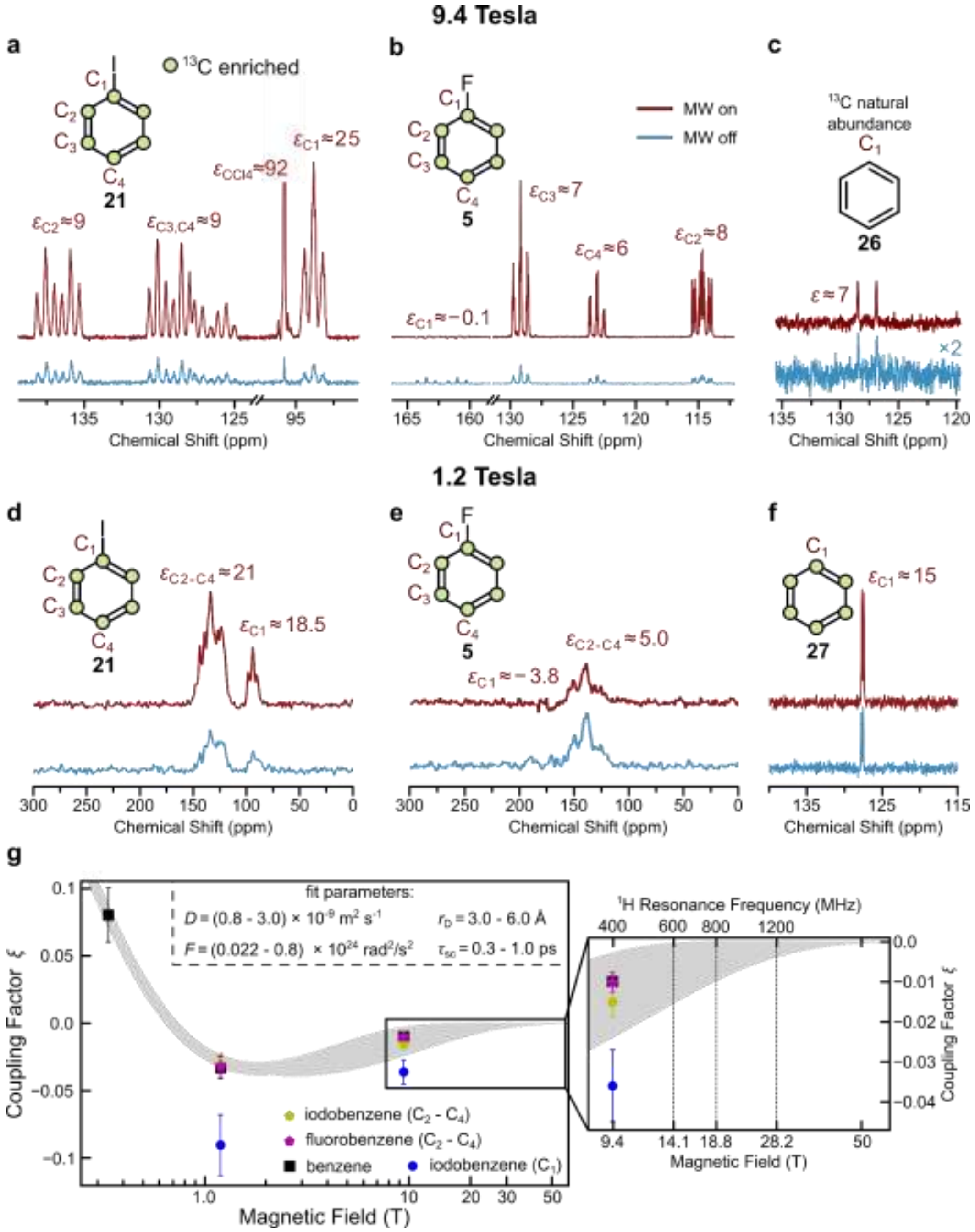

Supplementary Figure 23: (a-c) ^{13}C DNP NMR spectra (red) and Boltzmann spectra (blue) at 9.4 T with CCl_4 as a solvent (c(PA) ≈ 10 – 25 mM) of (a) iodobenzene-$^{13}C_6$ **21** (NS (number of scans): NS_{DNP} = 8, NS_{Boltz} = 32), (b) fluorobenzene-$^{13}C_6$ **5** (NS_{DNP} = 32, NS_{Boltz} = 32, ^1H decoupling), and (c) benzene **26** (NS_{DNP} = 16, NS_{Boltz} = 128). Spectra were scaled to the same SNR and were initially reported in ref.[16], (d-f) ^{13}C DNP NMR spectra at 1.2 T of neat solutions (d) iodobenzene-$^{13}C_6$ **21** (NS_{DNP} = 1, NS_{Boltz} = 56), (e) fluorobenzene-$^{13}C_6$ **5** (NS_{DNP} = 1, NS_{Boltz} = 56), and (f) benzene-$^{13}C_6$ **27** (NS_{DNP} = 4, NS_{Boltz} = 240) with c ≈ 10 mM TEMPONE-^{15}N-d_{16} measured according to protocols from the literature.[6,9,13] (g) Calculated magnetic field dependence of the coupling factor (grey area) according to eq. 6 with D =

$D_r + D_s$ (Supplementary equations 1) and $F = \frac{4\pi^2 \langle A_i \rangle^2}{\tau_{\mathrm{p},i}}$ from Supplementary equation 9. Experimental coupling factors are listed in Supplementary Tables 7 and 8. ξ(benzene) at 0.34 T was taken from the literature[39] and ξ of C_1 of iodobenzene (blue circles) was neglected for the simulation, due to lack of low field data. Zoom-in on the high field region of the simulation highlights state-of-the-art NMR resonance frequencies. Estimated error bars of ξ are up to 25%. Source data are provided as a Source Data file.[43]

Supplementary Discussion 3: Sensitivity comparison of 1D and 2D DNP with commercial probes

NMR line width (LW) and SNR were determined for the new DNP probe and compared to a commercial, standard bore liquid state 400 MHz NMR probe (Z116098, smart probe Bruker), in a magnet with a 20 shim coil system. Supplementary Table 9 lists these two parameters as obtained on standard Bruker reference samples (see table caption for sample composition).

Further ^{13}C line width comparisons were performed on a DNP sample of 200 mM $^{13}CHCl_3$ in CCl_4 doped with 10 mM TEMPONE-^{15}N-d_{16} in various sample arrangements (full tube and thin layer), in the DNP probe and the commercial liquid state probe, and with and without PA (Supplementary Table 10). Additionally, a quantitative sensitivity comparison between the DNP probe and the commercial NMR probe was performed for EAA-1,2,3,4-$^{13}C_4$ using the ^{13}C-^{13}C TOCSY sequence. The sample for the measurements with the DNP probe was specified in the main text (Fig.3 main text) and the same composition excluding PA was used for the measurements with the commercial probe. Here, in order to mimic routine NMR conditions, the PA was omitted in the measurements with the commercial probe. For the DNP measurements, a sample layer of ~40 µm corresponding to ~0.7 mg of target molecule was used, while for the measurements with the commercial probe either a sample layer of ~75 µm (~1.3 mg target molecule) or a full 5 mm NMR tube (~19 mg of target molecule) was employed. Acquisition and processing parameters were specified in the Methods and kept the same for all four measurements. In all measurements, a mixing time of $\tau_{\text{mix}} \approx$ 9.4 ms was used. 2D spectra are displayed in Supplementary Figure 24 and Fig.3 main text. The SNR was obtained from the 1D slices of the 2D spectra at two representative resonances of the EAA molecule (signals 2 and 4 in Fig.3 main text). They are depicted in Supplementary Figure 25 and the corresponding SNR values are tabulated in Supplementary Table 11.

To further compare our results with state-of-the-art NMR equipment, we performed a 2D INADEQUATE of p-cymene using a Bruker Prodigy nitrogen-cooled cryo-probe at 400 MHz. In general, these cryo-probes enable an SNR gain of a factor of 2-3 compared to commercial room temperature probes at the same resonance frequency. Experiments on the cryo-probe were performed with NS = 576 (~30 h acquisition time, Supplementary Figure 26) but otherwise identical experimental conditions as for the DNP 2D INADEQUATE presented in Fig.4 main text. We observe that the cryo-probe leads to an increased SNR of about a factor of 3 – 5 on the individual peaks as compared to the DNP experiment. This is consistent with the variation of the enhancement

factor over the target molecule and with the observation in Supplementary Table 9, that the ^{13}C sensitivity of our probe (no DNP) is about a factor of two worse than a commercial one.

Lastly, we also report T_{2n} values of p-cymene as a function of the PA concentration to give a perspective on the influence of paramagnetic compounds on the transverse relaxation time. Data shown in Supplementary tables 9 and 10 were partially reported in the PhD thesis of one of the authors.[16]

Supplementary Table 9: NMR line width (LW) at 50%, 0.55 %, and 0.11 % signal intensity, and SNR as obtained from the DNP probe and from a commercial Bruker probe (Z116098). ^{1}H Line shape measurements were performed on 3 % $CHCl_3$, 0.2 % tetramethylsilane (TMS) in acetone-d_6 (Bruker Z10230). ^{13}C line shape measurements were performed on 40 % dioxane in benzene-d_6 (American Society for Testing and Materials ASTM, sample named Bruker Z10163). SNR measurements were performed on 0.1 % ethylbenzene in $CDCl_3$ (Bruker Z10120) and Z10163 for ^{1}H and ^{13}C, respectively. All results were measured with sample rotation of ~10 – 20 Hz. Precision in LW is ± 0.3 Hz, uncertainty in SNR is 10 %. Table prepared with data extracted from spectra provided as source data files.[43]

Probe	nucleus	LW 50 % (Hz)	LW 0.55 % (Hz)	LW 0.11 % (Hz)	SNR
DNP	^{1}H	0.4	30	74	73
	^{13}C	0.5	21	36	130
Z116098	^{1}H	≤ 0.5	≤ 6	≤ 12	≥ 500
	^{13}C	≤ 0.2	≤ 2	≤ 4	≥ 200

Supplementary Table 10: ^{13}C NMR line width (LW) comparison of 200 mM $^{13}CHCl_3$ in CCl_4 with and without PA utilizing the DNP probe and a commercial NMR probe (Bruker Z116098). Measurements were performed on a full 5 mm NMR tube or on the thin layer DNP sample arrangement ($d \approx 75$ µm). (*) Sample composition was 90/10 $CCl_4/CHCl_3$ (v/v) (see Supplementary Figure 4). (**) Measurement performed after further optimization NMR conditions. Precision in LW is ± 0.3 Hz. Table prepared with data extracted from spectra provided as source data files.[43]

Probe	*c*(TEMPONE-^{15}N-d_{16}) (mM)	sample arrangement	microwave irradiation	LW 50 % (Hz) $^{13}CCl_4$	LW 50 % (Hz) $^{13}CHCl_3$
DNP	10	thin layer	no	3.8	15.5
	10	thin layer	yes	6.9	16.6
	–	thin layer	no	5.5*	5.2
	–	thin layer	yes	18*	5.0
	10	full tube	no	3.5	8.5
	–	full tube	no	3.8	2.6
Z116098	10	thin layer	no	3.7	13.5
	–	thin layer	no	2.8	2.1
	10	full tube	no	2.1	7.4
	–	full tube	no	2.0	1.6

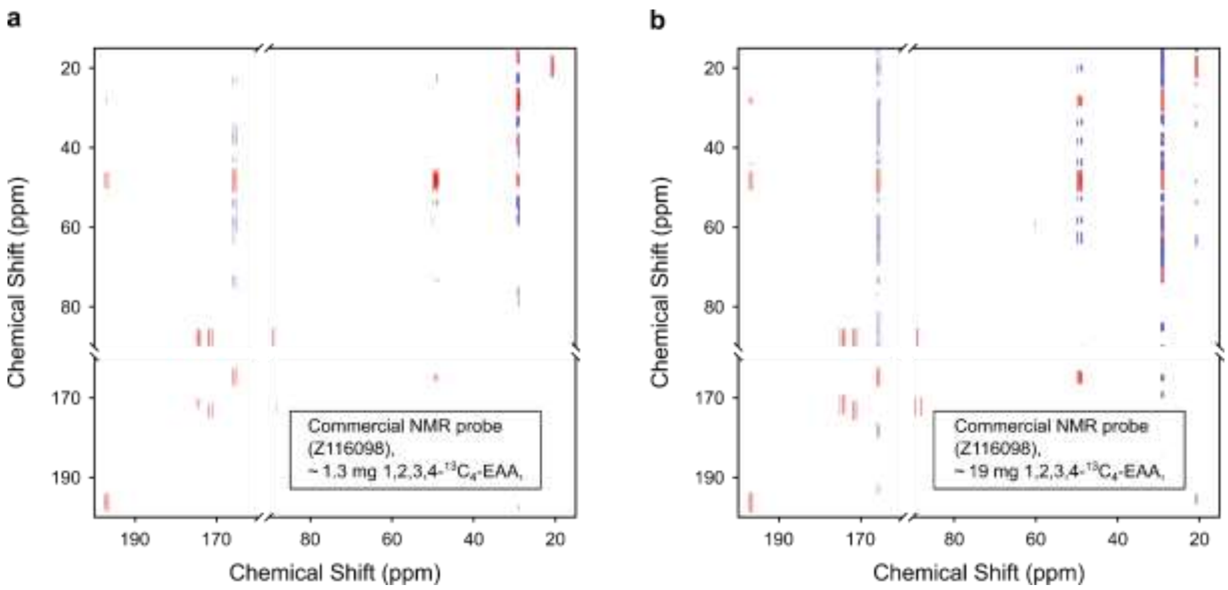

Supplementary Figure 24: (a-b) 2D ^{13}C-TOCSY of 500 mM 1,2,3,4-$^{13}C_4$-EAA in CCl_4 experiment in a commercial NMR probe for sensitivity comparison. (a) Spectra obtained in a sample arrangement of a thin layer; (b) spectra in a full NMR tube. Both spectra were recorded with 8 scans or 2 hours acquisition time. Red and blue contours indicate positive and negative values, respectively. Source data are provided as a Source Data file.[43]

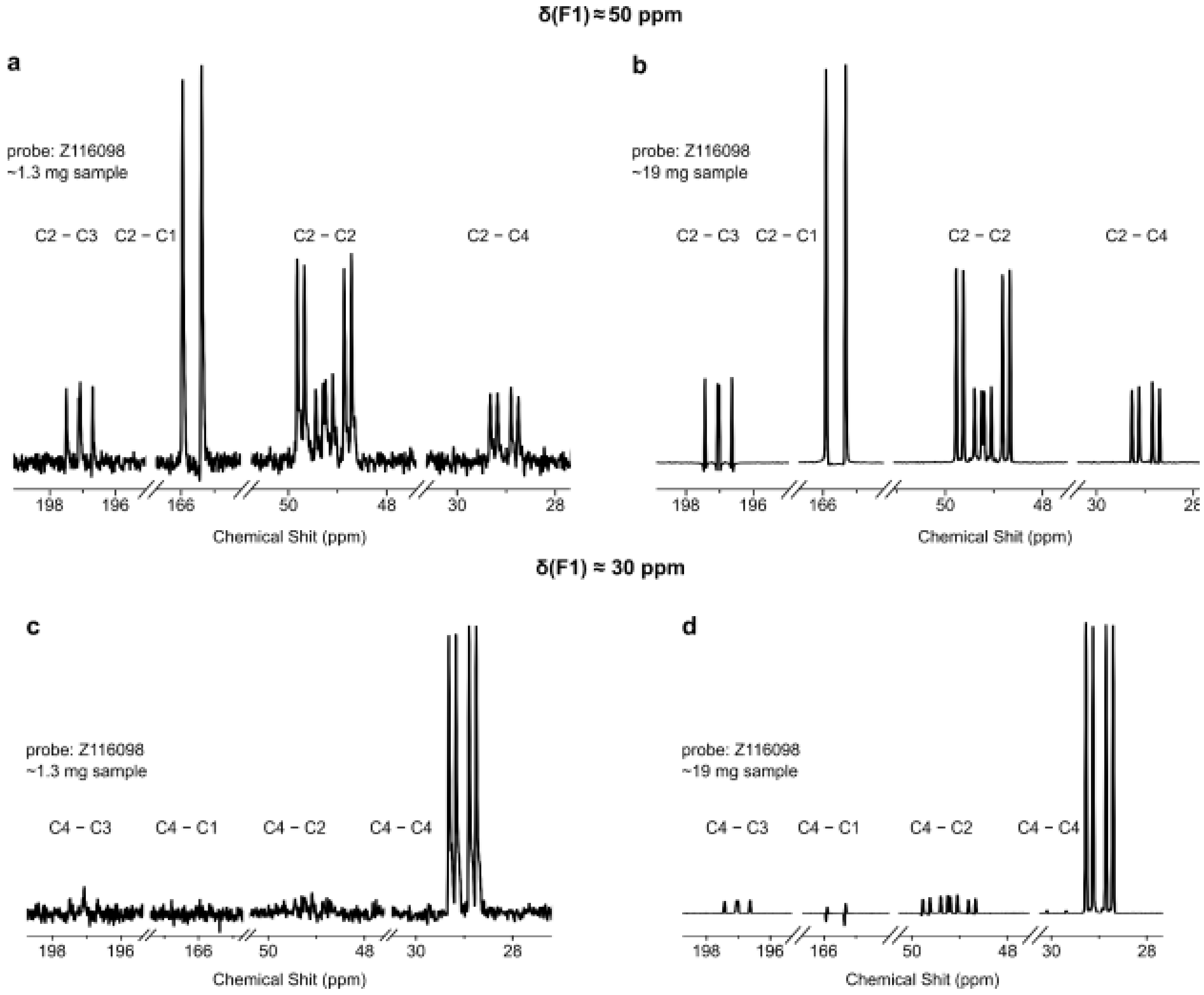

Supplementary Figure 25: 1D slice at $\delta(\mathrm{F1}) \approx 50$ ppm (a-b) and at $\delta(\mathrm{F1}) \approx 30$ ppm (c-d) of the 2D ^{13}C-TOCSY spectra of ethyl acetoacetate-1,2,3,4-$^{13}C_4$ (0.5 M in CCl_4) measured in the commercial Z116098 Bruker probe with two different sample volumes. 1D slices of the corresponding DNP measurements are depicted in Supplementary Figure 11c-d. SNR are reported in Supplementary Table 11.[43]

Supplementary Table 11: SNR of 2D ^{13}C-DNP-TOCSY spectra of ethyl acetoacetate-1,2,3,4-$^{13}C_4$ (0.5 M in CCl_4) measured with the DNP probe and the commercial Z116098 Bruker probe under similar experimental time (8 scans, 2 h under DNP as well as 16 scans, 4 hours without DNP). SNR were evaluated based on 1D slices at specific chemical shift values along the indirect (F1) dimension, here ~50 ppm and ~30 ppm corresponding to C2 and C4 of ethyl acetoacetate-1,2,3,4-$^{13}C_4$. SNR were calculated using Topspin 3.6.1. Errors in SNR vary depending on the signal intensities, between 10% and 20%. We note that when comparing SNR in the DNP probe (MW off) versus the commercial probe, the DNP sample contains the PA, while the standard sample does not. This affects relaxation times, line widths and thus the observed SNR. Therefore, the SNR might not directly correspond to the enhancement, as the line width is variably affected by the DNP depending on the chemical site. Table prepared with data extracted from spectra provided as source data files.[43]

Probe	Sample (mg)	Signal	ε (from peak integral)	SNR (from peak height)
DNP probe (MW on)	0.7	C2 – C1	13	316
		C2 – C2	11	168
		C2 – C3	9	9.2
		C2 – C4	12	22.3
DNP probe (MW off)	0.7	C2 – C1		33
		C2 – C2		15
		C2 – C3		5.5
		C2 – C4		7.0
Commercial probe Z116098	1.3	C2 – C1		47.9
		C2 – C2		21.4
		C2 – C3		9.4
		C2 – C4		7.6
Commercial probe Z116098	19	C2 – C1		973
		C2 – C2		493
		C2 – C3		209
		C2 – C4		205
DNP probe (MW on)	0.7	C4 – C1	9	9.2
		C4 – C2	9	29.4
		C4 – C3	9	7.1
		C4 – C4	8	119
DNP probe (MW off)	0.7	C4 – C1		2.7
		C4 – C2		3.7
		C4 – C3		4.0
		C4 – C4		51
Commercial probe Z116098	1.3	C4 – C1		2
		C4 – C2		2.5
		C4 – C3		3.5
		C4 – C4		34
Commercial probe Z116098	19	C4 – C1		30.7
		C4 – C2		62.0
		C4 – C3		40.9
		C4 – C4		974

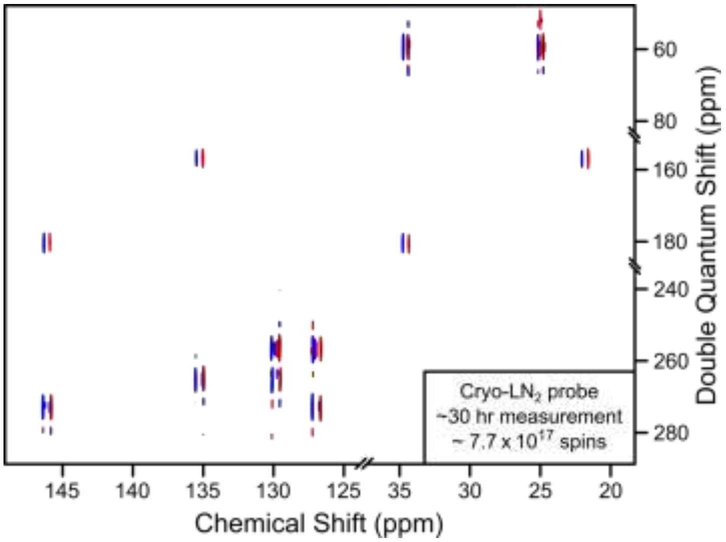

Supplementary Figure 26: 2D INADEQUATE of p-cymene obtained using a LN_2 cryo-probe at 400 MHz under otherwise identical experimental conditions (sample composition, tube-in-tube setup, and RF parameters) as described in Fig.4 main text. The only exception was that here the number of scans was NS = 576 (~30 h acquisition time). The SNR was ~15 – 16. Red and blue contours indicate positive and negative values, respectively. Source data are provided as a Source Data file.[43]

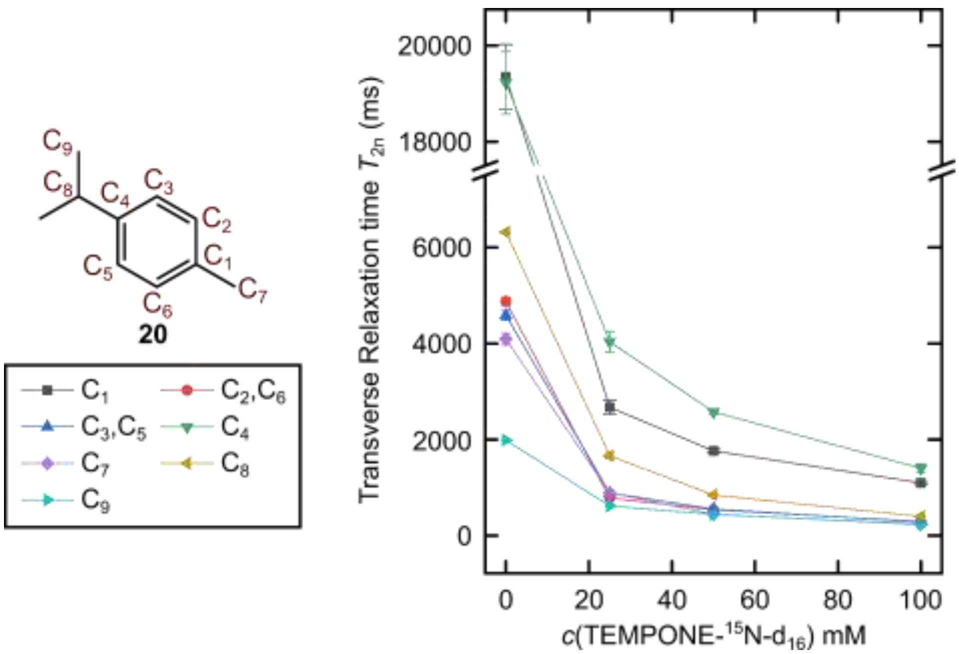

Supplementary Figure 27: Transverse nuclear relaxation times (T_{2n}) of p-cymene at different PA concentrations, determined using the standard Carr-Purcell-Meiboom-Gill (CPMG) pulse sequence implemented in Topspin 4.14. Data analysis was performed with Topspin 4.14. The uncertainty was determined from the standard deviation of the fit and varies from ~0.5 % to ~7% of the experimental value. Experimental conditions were comparable to the conditions in Fig.4 main text. Source data are provided as a Source Data file.[43]

Supplementary References

1 Bennati, M. & Orlando, T. Overhauser DNP in Liquids on ^{13}C Nuclei. *eMagRes* **8**, 11-18 (2019).

2 Hausser, K.-H. & Stehlik, D. Dynamic Nuclear Polarization in Liquids. *Adv. Magn. Reson.* **3**, 79-139 (1968).

3 Solomon, I. Relaxation Processes in a System of Two Spins. *Phys. Rev.* **99**, 559-565 (1955).

4 Keeler, J. *Understanding NMR Spectroscopy*. 2 edn, (John Wiley & Sons, Ltd. Chichester, 2010).

5 Türke, M. T., Parigi, G., Luchinat, C. & Bennati, M. Overhauser DNP with ^{15}N labelled Fremy's salt at 0.35 Tesla. *Phys. Chem. Chem. Phys.* **14**, 502-510 (2012).

6 Türke, M. T. & Bennati, M. Saturation factor of nitroxide radicals in liquid DNP by pulsed ELDOR experiments. *Phys. Chem. Chem. Phys.* **13**, 3630-3633 (2011).

7 Polnaszek, C. F. & Bryant, R. G. Nitroxide radical induced solvent proton relaxation: Measurement of localized translational diffusion. *J. Chem. Phys.* **81**, 4038-4045 (1984).

8 Hwang, L.-P. & Freed, J. H. Dynamic effects of pair correlation functions on spin relaxation by translational diffusion in liquids. *J. Chem. Phys.* **63**, 4017-4025 (1975).

9 Orlando, T. *et al.* Dynamic Nuclear Polarization of ^{13}C Nuclei in the Liquid State over a 10 Tesla Field Range. *Angew. Chem. Int. Ed.* **58**, 1402-1406 (2019).

10 Müller-Warmuth, W., Vilhjalmsson, R., Gerlof, P. A. M., Smidt, J. & Trommel, J. Intermolecular interactions of benzene and carbon tetrachloride with selected free radicals in solution as studied by ^{13}C and ^{1}H dynamic nuclear polarization. *Mol. Phys.* **31**, 1055-1067 (1976).

11 Noack, F., Krüger, G. J., Müller-Warmuth, W. & van Steenwinkel, R. Stochastische Prozesse in Spinsystemen. *Z. Naturforsch. A* **22**, 2102-2108 (1967).

12 Orlando, T., Kuprov, I. & Hiller, M. Theoretical analysis of scalar relaxation in ^{13}C-DNP in liquids. *J. Magn. Reson. Open* **10-11,** 100040 (2022).

13 Levien, M., Hiller, M., Tkach, I., Bennati, M. & Orlando, T. Nitroxide Derivatives for Dynamic Nuclear Polarization in Liquids: The Role of Rotational Diffusion. *J. Phys. Chem. Lett.* **11**, 1629-1635 (2020).

14 Bloch, F. Nuclear Induction. *Phys. Rev.* **70**, 460-474 (1946).

15 Enkin, N. *et al.* A high saturation factor in Overhauser DNP with nitroxide derivatives: the role of ^{14}N nuclear spin relaxation. *Phys. Chem. Chem. Phys.* **17**, 11144-11149 (2015).

16 Levien, M. Development of ^{13}C Liquid State Dynamic Nuclear Polarization at 9.4 Tesla, *PhD Thesis, Göttingen* (2023); doi:10.53846/goediss-9997.

17 Maly, T. *et al.* Dynamic nuclear polarization at high magnetic fields. *J. Chem. Phys.* **128,** 052211 (2008).

18 Shaka, A. J., Lee, C. J. & Pines, A. Iterative schemes for bilinear operators; application to spin decoupling. *J. Magn. Reson.* **77**, 274-293 (1988).

19 Herb, K., Tschaggelar, R., Denninger, G. & Jeschke, G. Double resonance calibration of g factor standards: Carbon fibers as a high precision standard. *J. Magn. Reson.* **289**, 100-106 (2018).

20 Carrington, A. & McLachlan, A. D. *Introduction to Magnetic Resonance*. (Harper and Row, 1967).

21 Poole, C. P. *Electron Spin Resonance: A Comprehensive Treatise on Experimental Techniques*. Vol. 2 (Wiley, 1969).

22 Prandolini, M. J., Denysenkov, V. P., Gafurov, M., Endeward, B. & Prisner, T. F. High-field dynamic nuclear polarization in aqueous solutions. *J. Am. Chem. Soc.* **131**, 6090-6092 (2009).

23 Sezer, D., Gafurov, M., Prandolini, M. J., Denysenkov, V. P. & Prisner, T. F. Dynamic nuclear polarization of water by a nitroxide radical: rigorous treatment of the electron spin saturation and comparison with experiments at 9.2 Tesla. *Phys. Chem. Chem. Phys.* **11**, 6638-6653 (2009).

24 Biller, J. R. *et al.* Relaxation times and line widths of isotopically-substituted nitroxides in aqueous solution at X-band. *J. Magn. Reson.* **212**, 370-377 (2011).

25 Schweiger, A. & Jeschke, G. *Principles of Pulsed Electron Paramagnetic Resonance*. (Oxford University Press, 2001).

26 Liu, G. *et al.* One-thousand-fold enhancement of high field liquid nuclear magnetic resonance signals at room temperature. *Nat. Chem.* **9**, 676-680 (2017).

27 Biller, J. R. *et al.* Frequency dependence of electron spin relaxation times in aqueous solution for a nitronyl nitroxide radical and perdeuterated-tempone between 250 MHz and 34 GHz. *J. Magn. Reson.* **225**, 52-57 (2012).

28 Biller, J. R. *et al.* Electron spin-lattice relaxation mechanisms of rapidly-tumbling nitroxide radicals. *J. Magn. Reson.* **236**, 47-56 (2013).

29 Froncisz, W. *et al.* Saturation recovery EPR and ELDOR at W-band for spin labels. *J. Magn. Reson.* **193**, 297-304 (2008).

30 Windle, J. J. Hyperfine coupling constants for nitroxide spin probes in water and carbon tetrachloride. *J. Magn. Reson.* **45**, 432-439 (1981).
31 Neese, F. The ORCA program system. *WIREs Comput. Mol. Sci.* **2**, 73-78 (2011).
32 Rezac, J. & Hobza, P. Benchmark Calculations of Interaction Energies in Noncovalent Complexes and Their Applications. *Chem. Rev.* **116**, 5038-5071 (2016).
33 Tomasi, J., Mennucci, B. & Cammi, R. Quantum mechanical continuum solvation models. *Chem. Rev.* **105**, 2999-3093 (2005).
34 Kutzelnigg, W., Fleischer, U. & Schindler, M. Vol. 23 Ch. The IGLO-Method: Ab Initio Calculation and Interpretation of NMR Chemical Shifts and Magnetic Susceptibilities, (Springer-Verlag, 1990).
35 Hess, B. A. Applicability of the no-pair equation with free-particle projection operators to atomic and molecular structure calculations. *Phys. Rev. A* **32**, 756-763 (1985).
36 Douglas, M. & Kroll, N. M. Quantum electrodynamical corrections to the fine structure of helium. *Ann. Phys.* **82**, 89-155 (1974).
37 Pantazis, D. A., Chen, X. Y., Landis, C. R. & Neese, F. All-Electron Scalar Relativistic Basis Sets for Third-Row Transition Metal Atoms. *J. Chem. Theory. Comput.* **4**, 908-919 (2008).
38 Kucuk, S. E. & Sezer, D. Multiscale computational modeling of ^{13}C DNP in liquids. *Phys. Chem. Chem. Phys.* **18**, 9353-9357 (2016).
39 Wang, X. *et al.* Optimization and prediction of the electron-nuclear dipolar and scalar interaction in ^{1}H and ^{13}C liquid state dynamic nuclear polarization. *Chem. Sci.* **6**, 6482-6495 (2015).
40 Ertl, H. & Dullien, F. A. L. Self-diffusion and viscosity of some liquids as a function of temperature. *Angew. Chem. Int. Ed.* **19**, 1215-1223 (1973).
41 Dubroca, T., Wi, S., van Tol, J., Frydman, L. & Hill, S. Large volume liquid state scalar Overhauser dynamic nuclear polarization at high magnetic field. *Phys. Chem. Chem. Phys.* **21**, 21200-21204 (2019).
42 Dai, D. *et al.* Room-temperature dynamic nuclear polarization enhanced NMR spectroscopy of small biological molecules in water. *Nat. Commun.* **12**, 6880 (2021).
43 Levien, M. et al. Overhauser enhanced liquid state nuclear magnetic resonance spectroscopy in one and two dimensions. doi:10.25625/AQY3SI *Göttingen Research Online Database* (2024)
